# A reverse network pharmacology and bioinformatics-based approach to exploring medication patterns for polycystic ovary syndrome-related infertility

**DOI:** 10.3389/fmed.2025.1614165

**Published:** 2025-11-05

**Authors:** Yueyan Wang, Fan Jia, Jing Hu, Zhiqi Shi, Haixia Huang, Yahong Zhou

**Affiliations:** ^1^Department of Reproductive Medicine, Wuxi Affiliated Hospital of Nanjing University of Chinese Medicine, Wuxi, China; ^2^Institute of Traditional Chinese Medicine, Wuxi Affiliated Hospital of Nanjing University of Chinese Medicine, Wuxi, China; ^3^School of Medicine, Jiangnan University, Wuxi, China

**Keywords:** polycystic ovary syndrome, infertility, reverse network pharmacology, herbal formula prediction, traditional Chinese medicine

## Abstract

**Objective:**

To predict potential herbal medicines targeting polycystic ovary syndrome (PCOS)-related infertility using an in silico reverse network pharmacology approach and identify core herbal candidates.

**Methods:**

This computational study began by collecting disease targets for PCOS and infertility from multiple public databases. Common targets were identified, and active compounds associated with these targets were retrieved from the Uniprot and TCMSP databases. These compounds were subsequently filtered using PubChem and SwissADME based on pharmacokinetic properties and mapped to corresponding herbs via TCMSP. Herbal properties (nature, flavor, meridian tropism) were statistically analyzed. A core network of targets-compounds-herbs was constructed using Cytoscape 3.8.0, and a subnetwork was generated from nodes with a Degree > 20. Finally, Gene Ontology (GO) and Kyoto Encyclopedia of Genes and Genomes (KEGG) enrichment analyses were performed on the targets of the core herbal combination to elucidate potential mechanisms.

**Results:**

A total of 2,500 common targets for PCOS and infertility, 1,545 active compounds, and 488 corresponding herbs were identified. Analysis of herbal properties revealed a predominance of Warm and Pungent medicines, followed by Cold, Bitter, Neutral, and Sweet medicines. A core herbal combination consisting of *Ephedra sinica* (Mahuang), *Magnolia officinalis* (Houpo), *Bupleurum chinense* (Chaihu), *Chrysanthemum morifolium* (Juhua), *Angelica dahurica* (Baizhi), and *Morus alba* (Sangye) was identified through frequency statistics, association rules, and cluster analysis. GO and KEGG enrichment analyses of the core combination’s targets highlighted mechanisms involving oxidative stress, inflammatory responses, and endocrine regulation, including the TNF and PI3K-Akt signaling pathways.

**Conclusion:**

This study successfully employed reverse network pharmacology to predict a core herbal combination for treating PCOS-related infertility. The findings, while requiring experimental validation, offer novel insights for developing therapeutic strategies and provide a foundation for future clinical management.

## 1 Introduction

PCOS is a complex endocrine and metabolic disorder characterized by reproductive dysfunction and systemic metabolic disturbances, presenting high clinical heterogeneity across a woman’s lifespan from adolescence to menopause (1). Epidemiological studies indicate that PCOS predominantly affects women of reproductive age, with the highest prevalence observed between puberty and 40 years (2). Concurrently, infertility rates have risen globally; for instance, China experienced an increase from 12 to 18% between 2007 and 2020 (3). Notably, PCOS-associated infertility accounts for approximately 70% of anovulatory infertility cases, primarily driven by pathophysiological factors such as hyperandrogenism, insulin resistance, and chronic inflammation (4). Over the past decade, the incidence of PCOS in China has surged by 65%, which has further exacerbated its societal and healthcare burden (5).

Current clinical interventions for PCOS-related infertility are centered on ovulation induction (e.g., clomiphene citrate) and assisted reproductive technologies (ART). However, these approaches face significant limitations, including variable ovarian response rates, increased risks of multiple pregnancies, and substantial financial costs (6). In contrast, traditional Chinese medicine (TCM) demonstrates unique advantages in improving the ovarian microenvironment, regulating menstrual cycles, and enhancing pregnancy outcomes through its multi-target, systemic modulatory effects (7). Despite these benefits, the lack of standardized herbal protocols and insufficient mechanistic clarity has hindered its widespread clinical adoption and integration into mainstream medicine.

Network pharmacology, which integrates systems biology, bioinformatics, and data mining, has emerged as a powerful tool for elucidating the “multi-component, multi-target” mechanisms of herbal therapies (8). Unlike conventional methods that start with known herbs to identify their potential targets, reverse network pharmacology begins with disease-associated targets to trace back to bioactive compounds and their corresponding herbs. This “disease-target-drug” approach aligns well with TCM’s holistic philosophy of treating complex syndromes and offers a robust framework for discovering and optimizing herbal formulas.

Here, we employed a reverse network pharmacology strategy to predict herb-target interactions for PCOS-related infertility, analyze the properties of the identified herbs, and screen for core herbal combinations. This study aims to bridge the gap between traditional TCM theory and modern pharmacology, providing evidence-based, hypothesis-generating insights that could inform future therapeutic strategies for PCOS management.

## 2 Materials and methods

### 2.1 Acquisition of common targets for PCOS and infertility

The keyword “Polycystic Ovary Syndrome” was used to search the Genecards database (9) (v5.15),^1^ OMIM database (10) (Online Mendelian Inheritance in Man),^2^ PharmGKB database (11) (Pharmacogenomics Knowledgebase),^3^ DisGeNet database (12) (v7.0),^4^ and DrugBank database (13) (v5.1.10).^5^ Similarly, the keyword “Female infertility” was queried in the Genecards, OMIM, PharmGKB, DisGeNet, and TTD databases (14) (Therapeutic Target Database).^6^ All database searches were conducted in October 2023. After removing duplicate entries from each search, the intersection of PCOS and infertility targets was extracted. A Venn diagram was generated to visualize the overlapping targets.

### 2.2 Reverse identification of herbal compounds from targets

The list of common intersection targets was submitted to the Uniprot database (https://www.uniprot.org/, release 2023_04) to convert gene names into standardized protein names. The TCMSP database (Traditional Chinese Medicine Systems Pharmacology Database and Analysis Platform)^7^ was then used to retrieve all compounds associated with these target proteins. The SMILES (Simplified Molecular Input Line Entry System) identifiers of the retrieved compounds were obtained from the PubChem database.^8^ Active compounds were screened using the SwissADME web tool^9^ based on two criteria: “GI absorption = High” and “Druglikeness ≥ 2 Yes.” These widely accepted criteria were chosen to enrich for compounds with favorable oral bioavailability and pharmacokinetic properties, thereby increasing their potential as therapeutic agents.

### 2.3 Reverse mapping of herbs from active compounds

Herbs corresponding to the screened active compounds were identified using the TCMSP database. A comprehensive target-compound-herb network was constructed and visualized using Cytoscape software (v3.8.0). To identify the most influential components within this network, nodes (targets, compounds, and herbs) with a Degree value > 20 were defined as core components. The Degree > 20 threshold is a commonly used metric in network pharmacology to identify highly connected “hub” nodes that are considered functionally significant within the network. A subnetwork consisting of these core targets, compounds, and herbs was then extracted and visualized.

### 2.4 Analysis of herbal properties

The frequency, nature (Four Natures), flavor (Five Flavors), meridian tropism, and therapeutic categories of the herbs associated with the active compounds were statistically analyzed using Microsoft Excel. Herbs with a usage frequency > 10 were classified as core candidates for further analysis. Association rule mining was performed on these core herbs using IBM SPSS Modeler 18.0 software, with thresholds set at support > 1 and confidence > 60% to identify significant co-occurrence patterns. Finally, hierarchical cluster analysis of the core herbs was conducted with IBM SPSS Statistics 26.0 to identify distinct medication patterns and herbal clusters.

### 2.5 Screening of core herbal combinations and enrichment analysis

A core herbal combination was formulated based on a synthesis of the results from the frequency statistics, association rule analysis, and cluster analysis. The targets associated with this core herbal combination were retrieved from the TCMSP database. These targets were then intersected with the previously identified 2,500 common PCOS-infertility targets. To elucidate the underlying biological mechanisms of the core combination, Gene Ontology (GO) and Kyoto Encyclopedia of Genes and Genomes (KEGG) pathway enrichment analyses were performed on the final set of overlapping targets. The analysis was conducted using the DAVID (Database for Annotation, Visualization and Integrated Discovery) platform, and terms with an adjusted *p*-value (FDR) < 0.05 were considered statistically significant.

## 3 Results

### 3.1 Common targets of PCOS and infertility

**Figure 1:**
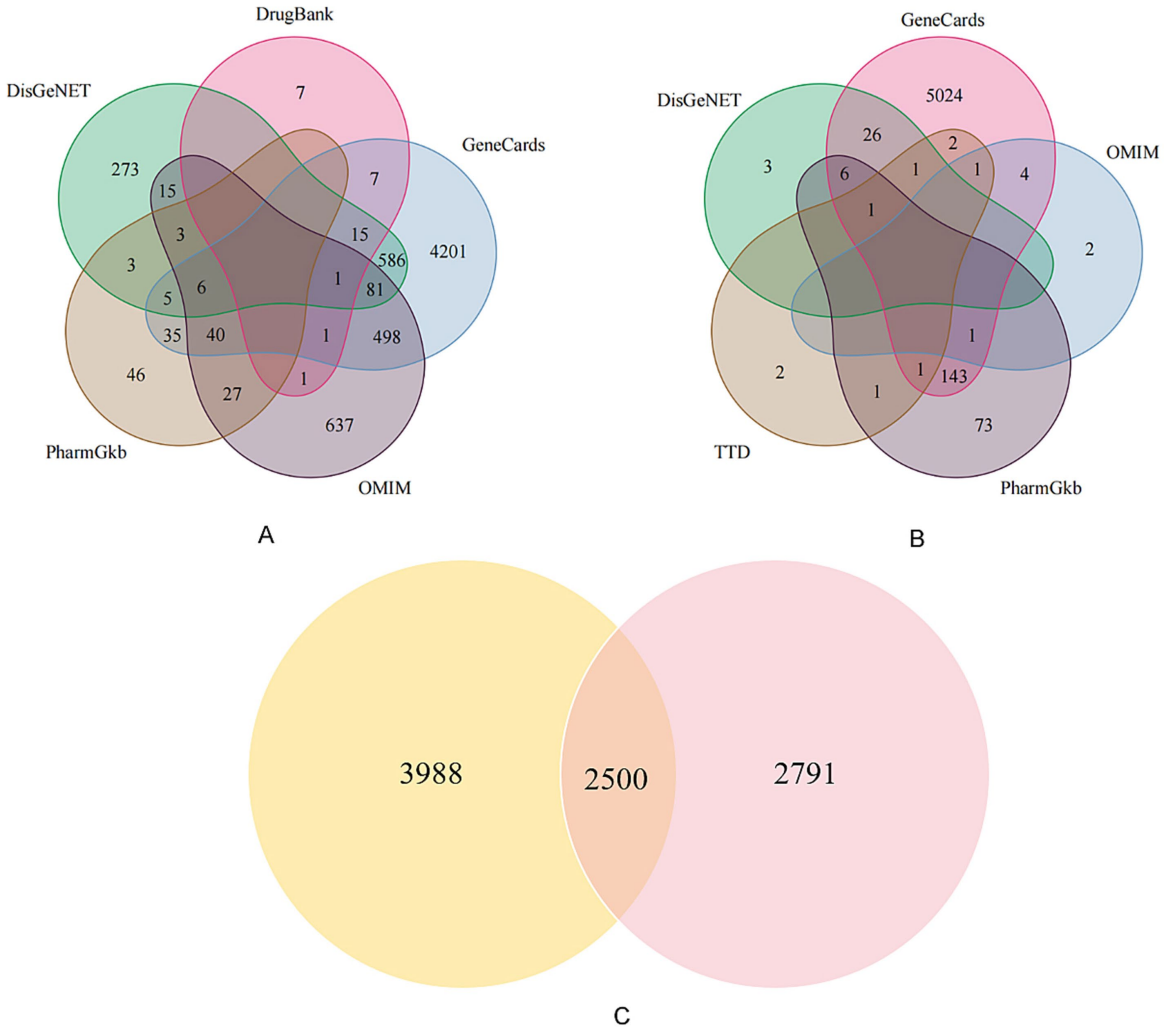
Venn diagrams of disease-related targets. This figure illustrates the process of identifying common targets. **(A)** Venn diagram showing the distribution of 6,488 unique PCOS-related targets collected from five databases: DrugBank, GeneCards, DisGeNET, PharmGkb, and OMIM. **(B)** Venn diagram showing the distribution of 5,291 unique infertility-related targets from five databases: GeneCards, OMIM, DisGeNET, TTD, and PharmGkb. **(C)** Venn diagram showing the 2,500 overlapping targets common to both PCOS and infertility, which formed the basis for subsequent analysis.

From the five databases, we retrieved 5,476 PCOS-related targets and 5,210 infertility-related targets from Genecards; 1,310 PCOS targets and 8 infertility targets from OMIM; 165 PCOS targets and 226 infertility targets from PharmGKB; 988 PCOS targets and 37 infertility targets from DisGeNet; and 32 PCOS targets from DrugBank. The TTD database provided an additional 9 infertility-related targets. After removing all duplicate entries, a total of 6,488 unique PCOS-related targets and 5,291 unique infertility-related targets were retained. The intersection of these two sets identified 2,500 shared targets, which were considered the common therapeutic targets for PCOS-related infertility (Figure 1).

### 3.2 Active compounds associated with PCOS-related infertility

The 2,500 common targets were reverse-mapped through the TCMSP database, resulting in the identification of 25,540 associated compounds. These compounds were then subjected to pharmacokinetic screening using PubChem and SwissADME with the criteria of “GI absorption = High” and “Druglikeness ≥ 2 Yes.” This filtering process yielded 1,545 active compounds predicted to have therapeutic potential for PCOS-related infertility (Table 1).

**Table 1:** Selection of top active compounds associated with PCOS-related infertility.

Chemical name	Mol ID	PubChem ID	GI absorption	Number of druglikeness “Yes” criteria
Quercetin	MOL000098	5,280,343	High	5
Kaempferol	MOL000422	5,280,863	High	5
17-beta-estradiol	MOL010919	5,757	High	5
(S)-Scoulerine	MOL000217	439,654	High	5
L-Bornyl acetate	MOL000196	93,009	High	4
Azeton	MOL004472	180	High	3
IPA	MOL008719	3,776	High	3
(L)-alpha-Terpineol	MOL000118	443,162	High	3
(R)-linalool	MOL000198	443,158	High	3
(2R)-2-methylbutan-1-ol	MOL009813	637,572	High	3
Propionic ether	MOL009843	7,749	High	3
Palmitic acid	MOL000069	985	High	2

A selection of active compounds is shown. The full list of 1,545 compounds is available in Supplementary Table S1.

### 3.3 Herbal medicines associated with PCOS-related infertility

**Figure 2:**
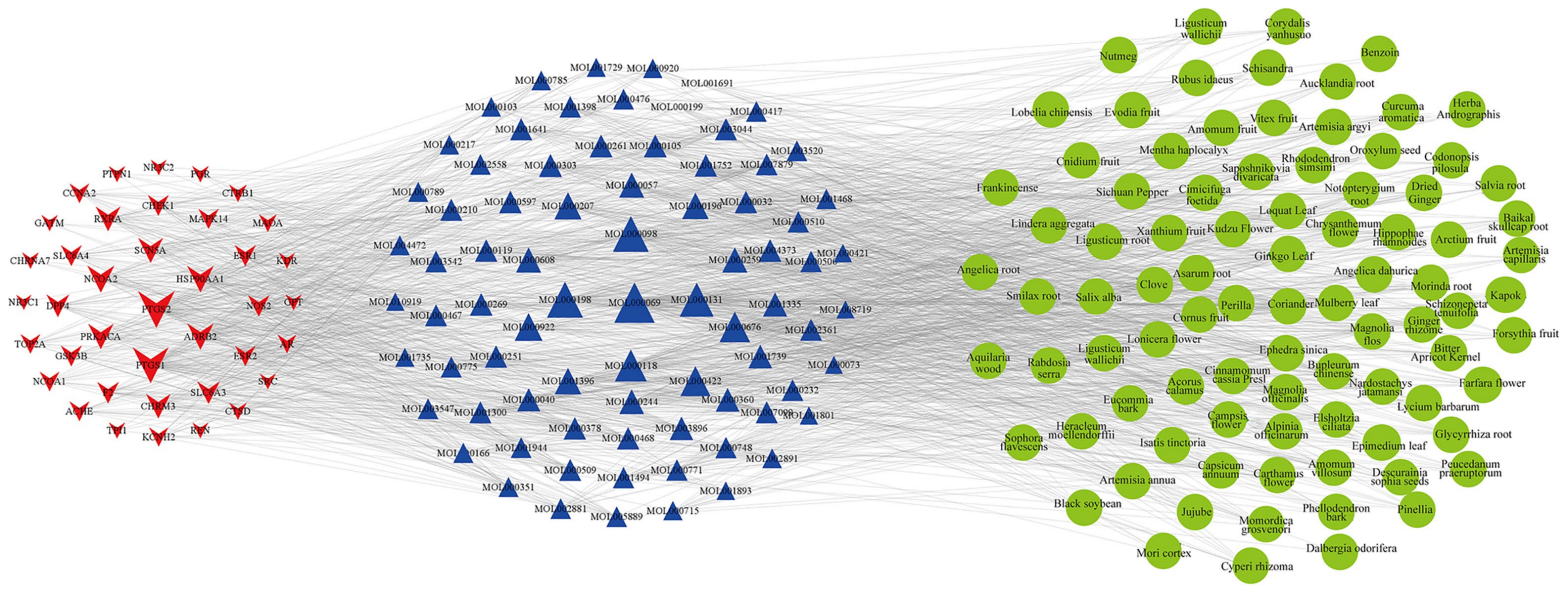
The core target-compound-herb network for PCOS-related infertility. This network illustrates the key interactions among disease targets, active compounds, and herbal medicines. Red star-shaped nodes represent the core PCOS-infertility targets, blue triangle nodes represent the core active compounds, and green circular nodes represent the core herbs. The size of each node is proportional to its Degree value, indicating its connectedness within the network. Larger nodes represent components with a higher number of connections, suggesting greater importance in the therapeutic mechanism.

The 1,545 active compounds were traced back to their herbal sources, corresponding to 488 unique herbs and a total of 180 targets. A preliminary target-compound-herb network was constructed using Cytoscape 3.8.0, which comprised 2,213 nodes and 15,465 edges. An analysis of the network topology revealed the top 10 herbs with the highest number of associated targets: *Pueraria lobata* (Gegen, 143 targets), *Morus alba* (Sangye, 136 targets), *Oroxylum indicum* (Muhudie, 135 targets), *Zanthoxylum bungeanum* (Huajiao, 126 targets), *Carthamus tinctorius* (Honghua, 121 targets), *Eriobotrya japonica* (Pipaye, 120 targets), *Ginkgo biloba* (Yinxingye, 120 targets), *Syzygium aromaticum* (Dingxiang, 119 targets), *Astragalus membranaceus* (Huangqi, 118 targets), and *Ephedra sinica* (Mahuang, 118 targets). To focus on the most significant interactions, nodes with a Degree > 20 (representing 40 targets, 79 compounds, and 89 herbs) were selected to reconstruct a refined, core target-compound-herb network (Figure 2).

### 3.4 Herbal property analysis

#### 3.4.1 Frequency analysis of herbs

Among the 488 herbs identified, 472 were validated against the *Chinese Pharmacopoeia* (2015) and *Chinese Herbal Medicine*, while 16 were excluded due to non-standardization. These herbs appeared a total of 6,365 times across the 1,545 compounds. High-frequency herbs (appearing >50 times) included *Ephedra sinica* (Mahuang, 91), *Glycyrrhiza uralensis* (Gancao, 71), *Cinnamomum cassia* (Guizhi, 64), *Bupleurum chinense* (Chaihu, 63), *Perilla frutescens* (Zisu, 59), *Coriandrum sativum* (Yansui, 59), *Chrysanthemum morifolium* (Juhua, 59), *Morus alba* (Sangye, 58), *Ginkgo biloba* (Yinxingye, 57), *Eriobotrya japonica* (Pipaye, 57), and *Asarum heterotropoides* (Xixin, 52). A total of 41 herbs exhibited frequencies >30 (Table 2).

**Table 2:** Statistical results of 41 Chinese herbal medicines with frequency >30 for PCOS-related infertility.

No.	Herb (Chinese name)	Latin name	Frequency	Percentage	No.	Herb (Chinese name)	Latin name	Frequency	Percentage
1	麻黄	*Ephedra sinica*	91	1.43%	22	川芎	Ligusticum chuanxiong	39	0.61%
2	甘草	Glycyrrhiza uralensis	71	1.12%	23	山茱萸	*Cornus officinalis*	39	0.61%
3	桂枝	*Cinnamomum cassia*	64	1.01%	24	金银花	*Lonicera japonica*	37	0.58%
4	柴胡	Bupleurum chinense	63	0.99%	25	石菖蒲	Acorus tatarinowii	37	0.58%
5	菊花	*Chrysanthemum morifolium*	59	0.93%	26	辣椒	*Capsicum annuum*	36	0.57%
6	芫荽	*Coriandrum sativum*	59	0.93%	27	青蒿	*Artemisia annua*	36	0.57%
7	紫苏	*Perilla frutescens*	59	0.93%	28	高良姜	*Alpinia officinarum*	35	0.55%
8	桑叶	*Morus alba*	58	0.91%	29	砂仁	Amomum villosum	35	0.55%
9	枇杷叶	*Eriobotrya japonica*	57	0.90%	30	香薷	Mosla chinensis	34	0.53%
10	银杏叶	*Ginkgo biloba*	57	0.90%	31	淫羊藿	Epimedium brevicornu	34	0.53%
11	细辛	Asarum heterotropoides	52	0.82%	32	黄柏	Phellodendron chinense	33	0.52%
12	生姜	*Zingiber officinale*	50	0.79%	33	降香	Dalbergia odorifera	33	0.52%
13	羌活	Notopterygium incisum	49	0.77%	34	满山红	Rhododendron dauricum	33	0.52%
14	辛夷	Magnolia biondii	47	0.74%	35	沙棘	*Hippophae rhamnoides*	33	0.52%
15	白芷	Angelica dahurica	43	0.68%	36	干姜	*Zingiber officinale*	32	0.50%
16	丹参	*Salvia miltiorrhiza*	43	0.68%	37	厚朴	Magnolia officinalis	32	0.50%
17	吴茱萸	Evodia rutaecarpa	43	0.68%	38	黄芩	Scutellaria baicalensis	32	0.50%
18	防风	Saposhnikovia divaricata	41	0.64%	39	凌霄花	*Campsis grandiflora*	32	0.50%
19	葛花	*Pueraria lobata*	41	0.64%	40	罗汉果	Siraitia grosvenorii	32	0.50%
20	杜仲	*Eucommia ulmoides*	40	0.63%	41	郁金	*Curcuma aromatica*	31	0.49%
21	板蓝根	Isatis indigotica	39	0.61%					

#### 3.4.2 Four natures, five flavors, and meridian tropism

**Figure 3:**
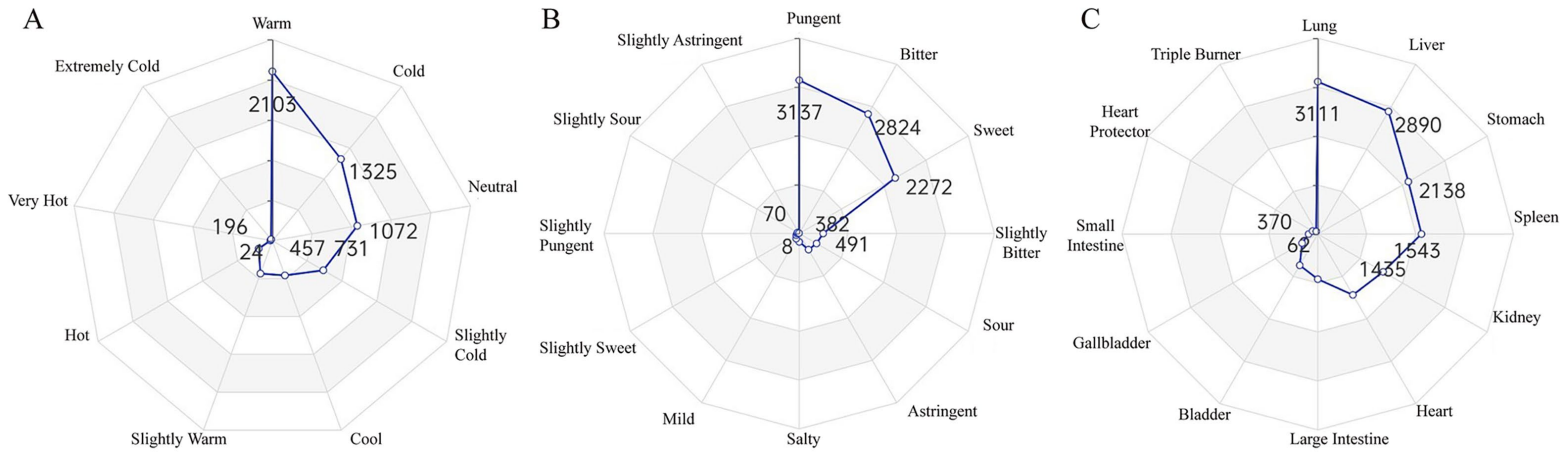
Analysis of herbal properties based on traditional chinese medicine theory. These radar charts quantify the TCM properties of the identified herbs. **(A)** Distribution of the Four Natures, showing a predominance of Warm and Cold properties. **(B)** Distribution of the Five Flavors, with Pungent, Bitter, and Sweet being the most common. **(C)** Distribution of Meridian Tropism, indicating that the herbs primarily act on the Lung, Liver, and Stomach meridians. The numbers on the axes represent the frequency of occurrence for each property.

Four Natures Analysis: The dominant medicinal properties were Warm (2,103 occurrences, 33.04%), Cold (1,325, 20.82%), and Neutral (1,072, 16.84%), followed by Cool, Slightly Cold, Slightly Warm, Hot, Very Hot, and Extremely Cold (Figure 3A).

Five Flavors Analysis: Pungent (3,137, 31.45%), Bitter (2,824, 28.31%), and Sweet (2,272, 22.78%) were the predominant flavors, with minor contributions from Slightly Bitter, Sour, Astringent, Salty, Mild, Slightly Sweet, Slightly Pungent, Slightly Sour, and Slightly Astringent (Figure 3B).

Meridian Tropism Analysis: The most frequently targeted meridians were the Lung meridian (3,111, 19.90%), Liver meridian (2,890, 18.48%), and Stomach meridian (2,138, 13.67%), followed by the Spleen (1,543, 9.87%), Kidney (1,543, 9.87%), and Heart meridians (1,435, 9.18%) (Figure 3C).

#### 3.4.3 Herbal categories

**Figure 4:**
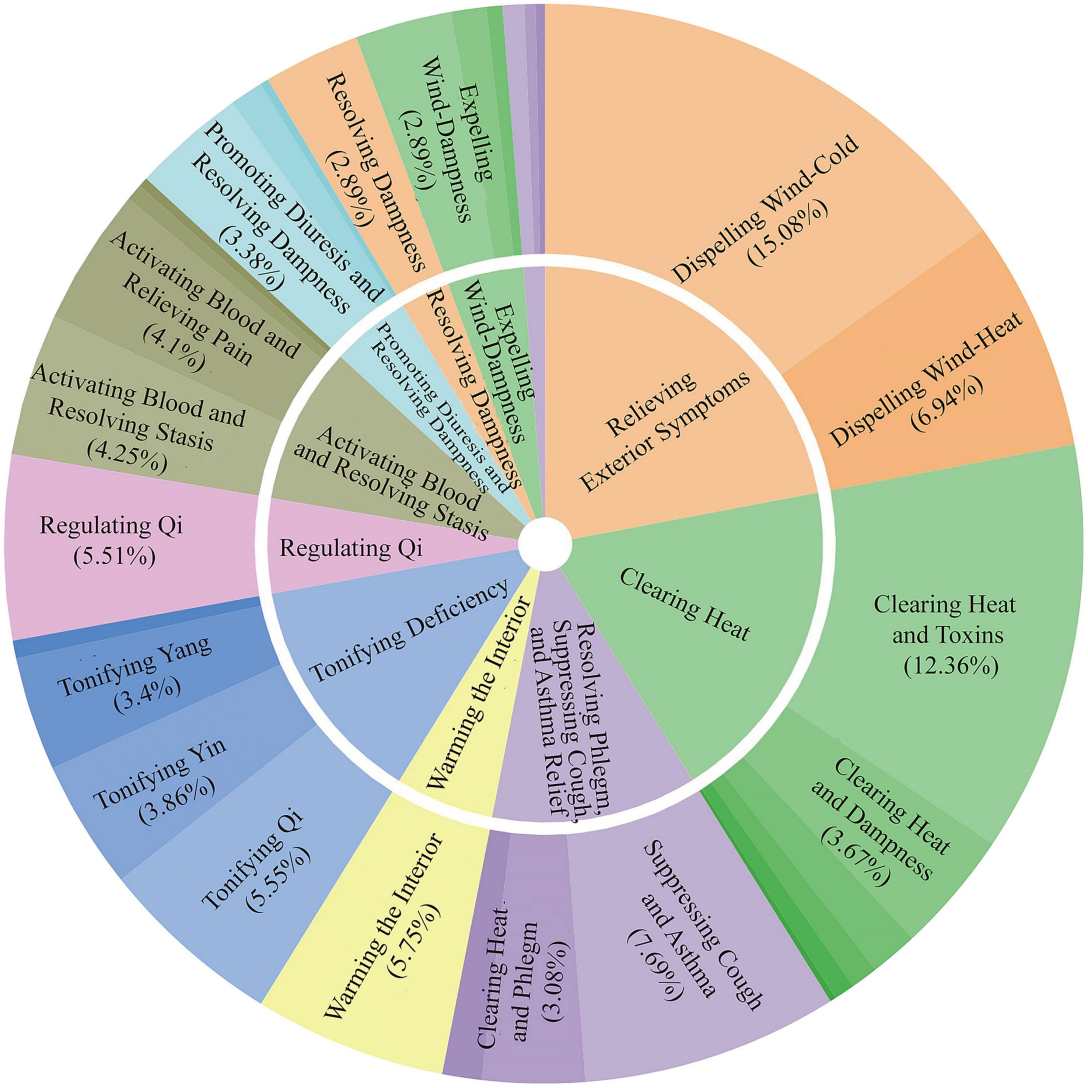
Sunburst chart of herbal therapeutic categories. This chart displays the hierarchical classification of the 488 herbs based on their primary therapeutic functions in TCM. The inner ring shows the main categories, while the outer ring provides more specific subcategories. The size of each segment corresponds to the percentage of herbs in that category, highlighting functions like Relieving Exterior Symptoms and Clearing Heat.

The identified herbs were classified into various therapeutic categories. The most prominent categories included Relieving Exterior Symptoms (15.08%), Clearing Heat (12.36%), and Tonifying Deficiency (5.55%). Subcategories further specified their actions, such as Dispelling Wind-Cold (a subcategory of Relieving Exterior), Clearing Heat and Toxins (a subcategory of Clearing Heat), and Suppressing Cough and Asthma (7.69%; Figure 4).

#### 3.4.4 Association rule analysis

**Figure 5:**
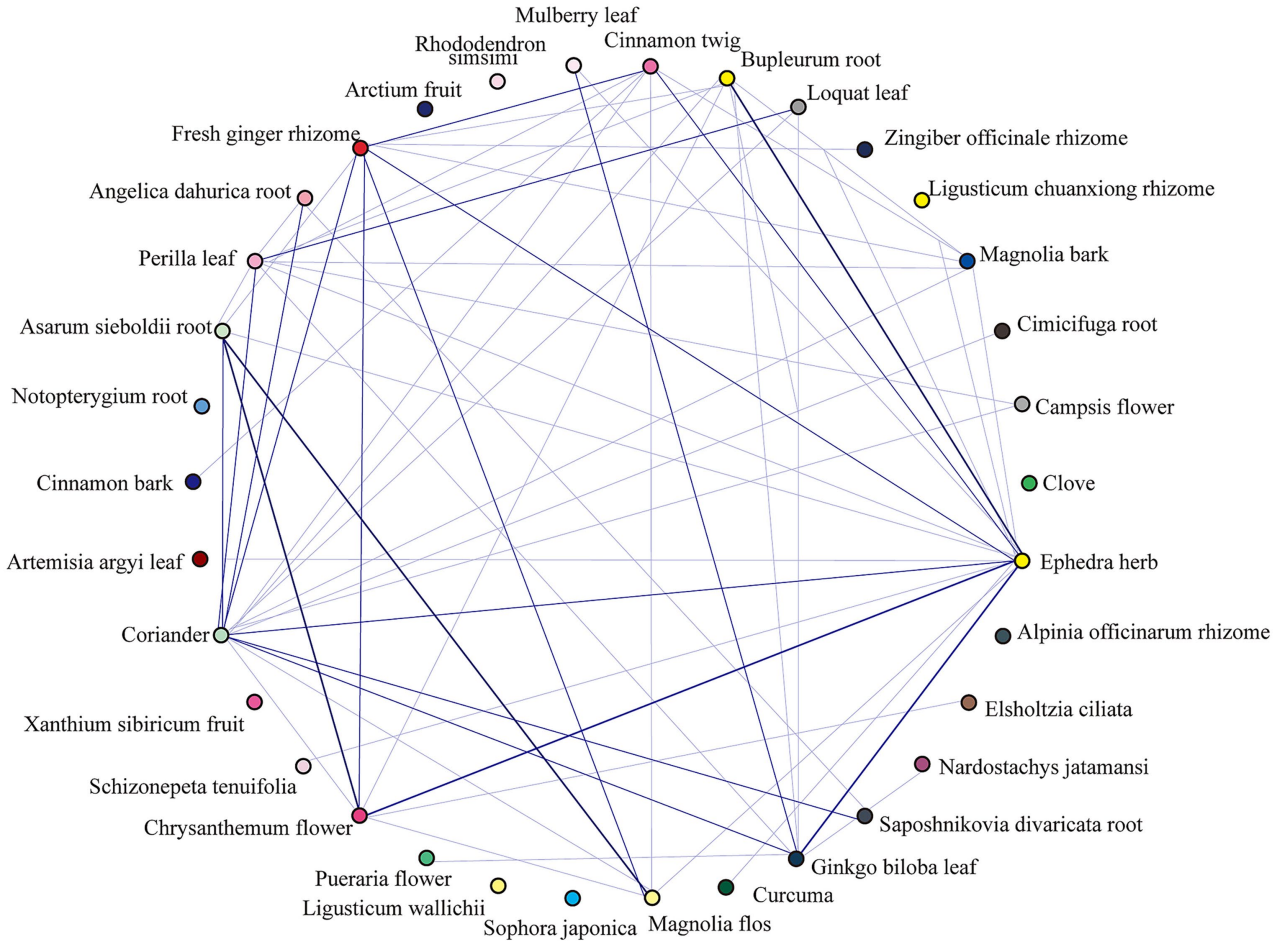
Chord diagram of top 30 herbal associations. This chord diagram visualizes the co-occurrence relationships among the top 30 most frequent herbs identified for PCOS-related infertility. Each herb is represented by an arc on the circumference. The connecting bands (chords) illustrate the strength of the association between pairs of herbs, with wider bands indicating a stronger co-occurrence based on the association rule analysis. This visualization highlights key herbal pairings, such as the strong relationship between *Ephedra sinica*, *Bupleurum chinense*, and *Chrysanthemum morifolium*.

A complex network analysis of 247 herbs (frequency ≥10) was performed using IBM SPSS Modeler 18.0, with thresholds set at “strong link = 19, weak link = 10.” This analysis revealed key herbal associations. For instance, a strong association was found where the presence of *Bupleurum chinense* (Chaihu) and *Ephedra sinica* (Mahuang) predicted the co-occurrence of *Chrysanthemum morifolium* (Juhua) (26 occurrences). The detailed binary and ternary association rules are presented in Tables 3, 4, and a visualization of the network is shown in Figure 5.

**Table 3:** Binary association rules of Chinese herbal medicines for PCOS-related infertility.

No.	Consequent (Chinese name)	Latin name	Antecedent (Chinese name)	Latin name	Frequency	Confidence	Lift
1	麻黄	*Ephedra sinica*	荆芥	Schizonepeta tenuifolia	24	79.17%	1.23
2	桂枝	*Cinnamomum cassia*	肉桂	*Cinnamomum aromaticum*	20	80.00%	1.04
3	芫荽	*Coriandrum sativum*	苍耳子	*Xanthium sibiricum*	21	71.43%	0.97
4	麻黄	*Ephedra sinica*	猫爪草	Ranunculus ternatus	16	62.50%	0.65

**Table 4:** Ternary association rules of Chinese herbal medicines for PCOS-related infertility.

No.	Consequent (Chinese name)	Latin name	Antecedent 1 (Chinese name)	Latin name	Antecedent 2 (Chinese name)	Latin name	Frequency	Confidence	Lift
1	菊花	*Chrysanthemum morifolium*	柴胡	Bupleurum chinense	麻黄	*Ephedra sinica*	26	61.54%	1.04
2	柴胡	Bupleurum chinense	菊花	*Chrysanthemum morifolium*	麻黄	*Ephedra sinica*	25	64.00%	1.04
3	麻黄	*Ephedra sinica*	菊花	*Chrysanthemum morifolium*	柴胡	Bupleurum chinense	19	84.21%	1.04
4	麻黄	*Ephedra sinica*	厚朴	Magnolia officinalis	柴胡	Bupleurum chinense	18	83.33%	0.97
5	柴胡	Bupleurum chinense	厚朴	Magnolia officinalis	麻黄	*Ephedra sinica*	17	88.24%	0.97
6	芫荽	*Coriandrum sativum*	细辛	Asarum heterotropoides	菊花	*Chrysanthemum morifolium*	23	60.87%	0.91
7	生姜	*Zingiber officinale*	芫荽	*Coriandrum sativum*	麻黄	*Ephedra sinica*	22	63.64%	0.91
8	麻黄	*Ephedra sinica*	紫苏	*Perilla frutescens*	芫荽	*Coriandrum sativum*	22	63.64%	0.91
9	紫苏	*Perilla frutescens*	芫荽	*Coriandrum sativum*	麻黄	*Ephedra sinica*	22	63.64%	0.91
10	厚朴	Magnolia officinalis	生姜	*Zingiber officinale*	麻黄	*Ephedra sinica*	21	66.67%	0.91
11	生姜	*Zingiber officinale*	细辛	Asarum heterotropoides	芫荽	*Coriandrum sativum*	21	66.67%	0.91
12	菊花	*Chrysanthemum morifolium*	细辛	Asarum heterotropoides	芫荽	*Coriandrum sativum*	21	66.67%	0.91
13	细辛	Asarum heterotropoides	菊花	*Chrysanthemum morifolium*	芫荽	*Coriandrum sativum*	21	66.67%	0.91
14	芫荽	*Coriandrum sativum*	生姜	*Zingiber officinale*	麻黄	*Ephedra sinica*	21	66.67%	0.91

### 3.5 Network pharmacological analysis of core herbal combination

#### 3.5.1 Screening of core herbal combination

**Figure 6:**
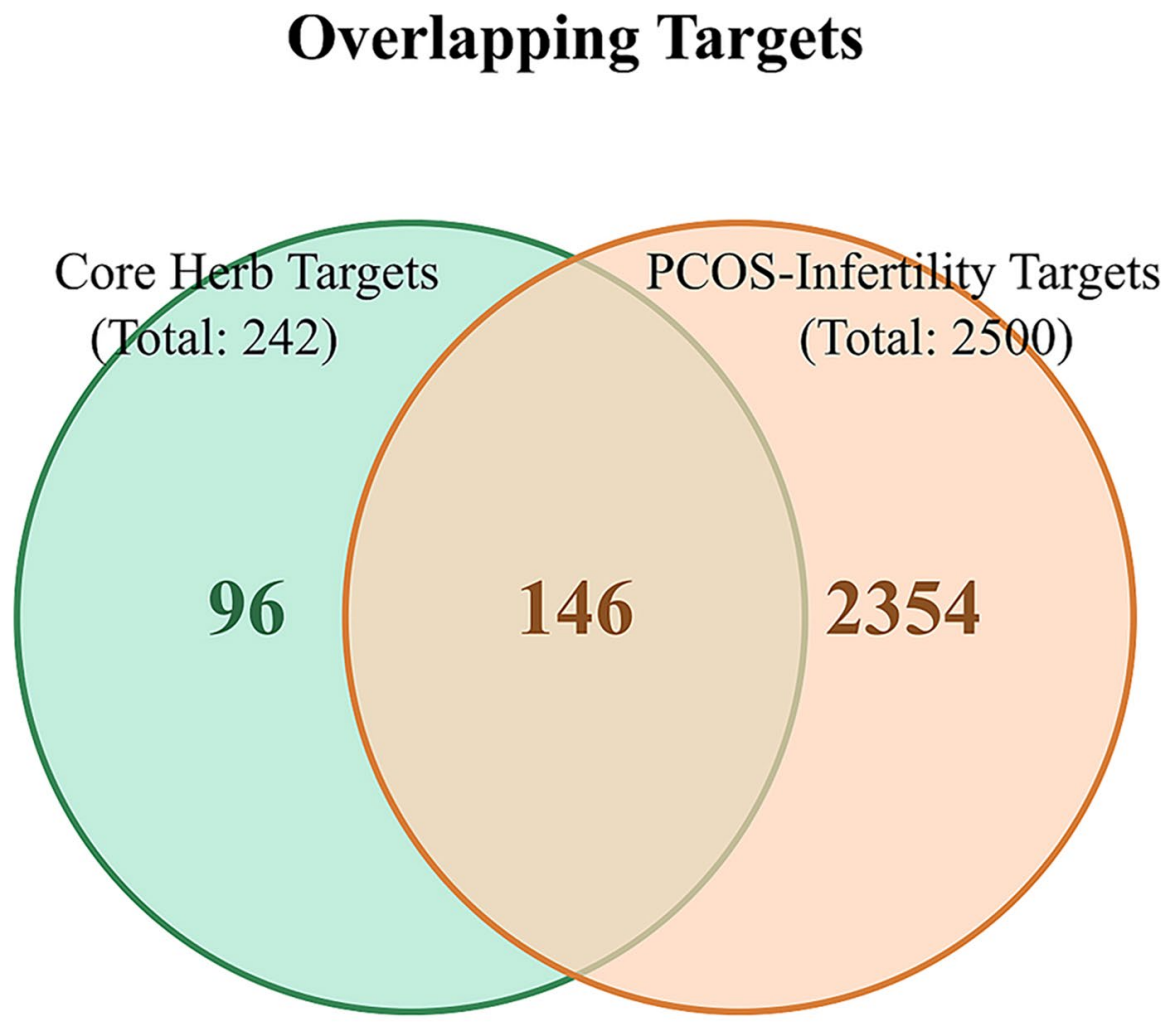
Venn diagram of overlapping targets between the core herbal combination and PCOS-infertility. The light green circle represents the 242 targets associated with the six core herbs. The light orange circle represents the 2,500 common targets of PCOS and infertility. The overlapping region indicates the 146 targets that are modulated by the core herbal combination and are also directly relevant to the pathophysiology of PCOS-related infertility.

Based on a comprehensive assessment of the frequency statistics, association rules, and cluster analysis, a core herbal combination was identified: “*Ephedra sinica* (Mahuang)–*Magnolia officinalis* (Houpo)–*Bupleurum chinense* (Chaihu)–*Chrysanthemum morifolium* (Juhua)–*Angelica dahurica* (Baizhi)–*Morus alba* (Sangye).” These six herbs contributed 23, 2, 17, 20, 23, and 29 active compounds, respectively. After removing duplicates, this combination yielded 55 unique active compounds, which corresponded to 242 potential targets. The intersection of these 242 targets with the 2,500 common PCOS-infertility targets resulted in 146 shared targets for the core combination (Figure 6).

#### 3.5.2 GO and KEGG enrichment analysis

**Figure 7:**
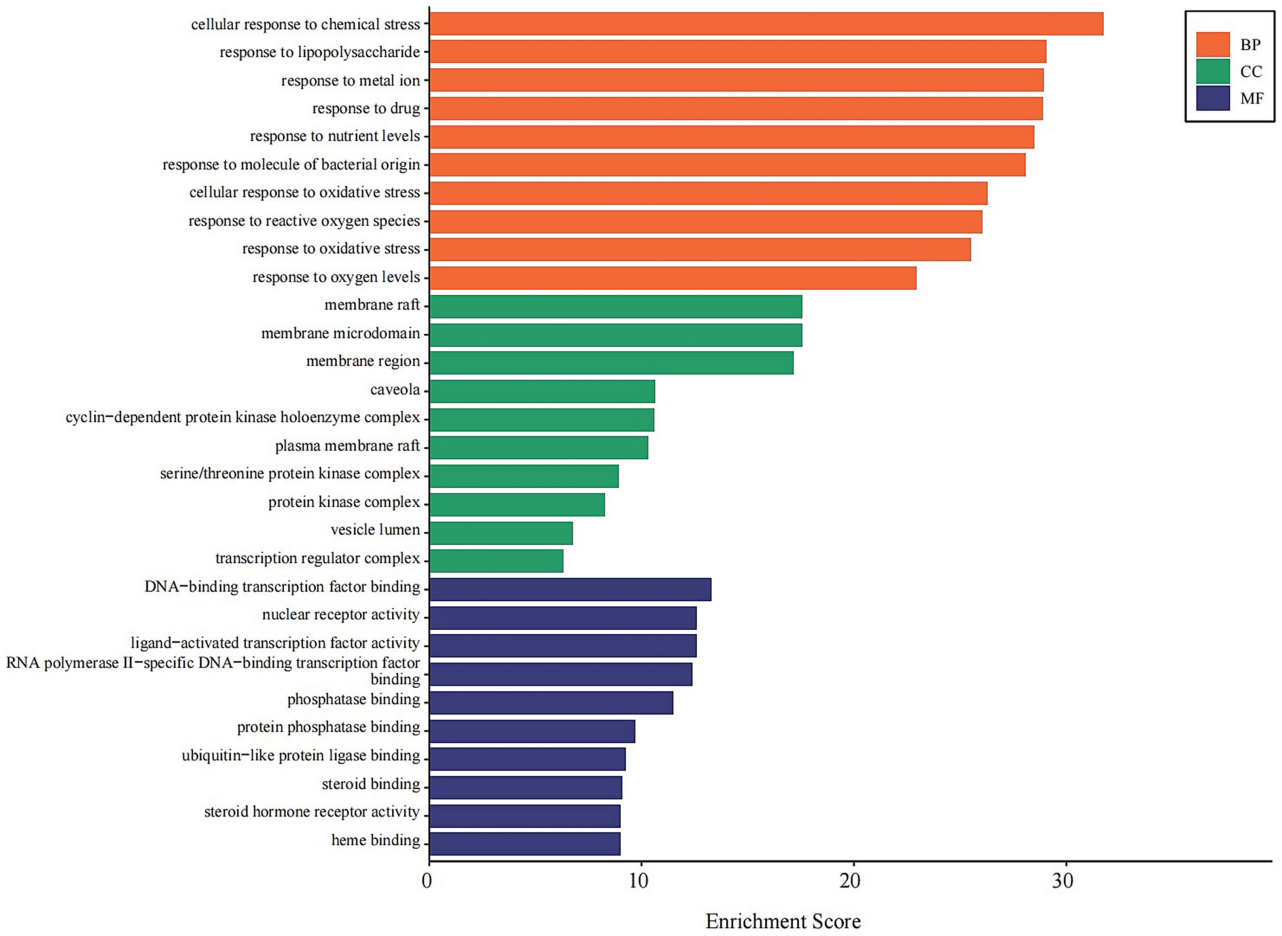
GO enrichment analysis of core combination targets. This bar chart displays the top 10 significantly enriched Gene Ontology (GO) terms for the 146 core targets, categorized by Biological Process (BP, orange), Cellular Component (CC, green), and Molecular Function (MF, blue). The x-axis represents the enrichment score, indicating the significance of each term. The analysis highlights the roles of these targets in stress responses, membrane signaling, and transcriptional regulation. Enriched terms with an adjusted *p*-value < 0.05 were considered significant.

GO Analysis: The 146 shared targets were subjected to GO enrichment analysis. The most significantly enriched biological processes (BP) included cellular response to chemical stress, response to lipopolysaccharide, and response to oxidative stress. Enriched cellular components (CC) primarily involved membrane raft, cyclin-dependent protein kinase holoenzyme complex, and serine/threonine protein kinase complex. For molecular functions (MF), key enriched terms included DNA-binding transcription factor binding, nuclear receptor activity, and steroid hormone receptor activity (Figure 7).

**Figure 8:**
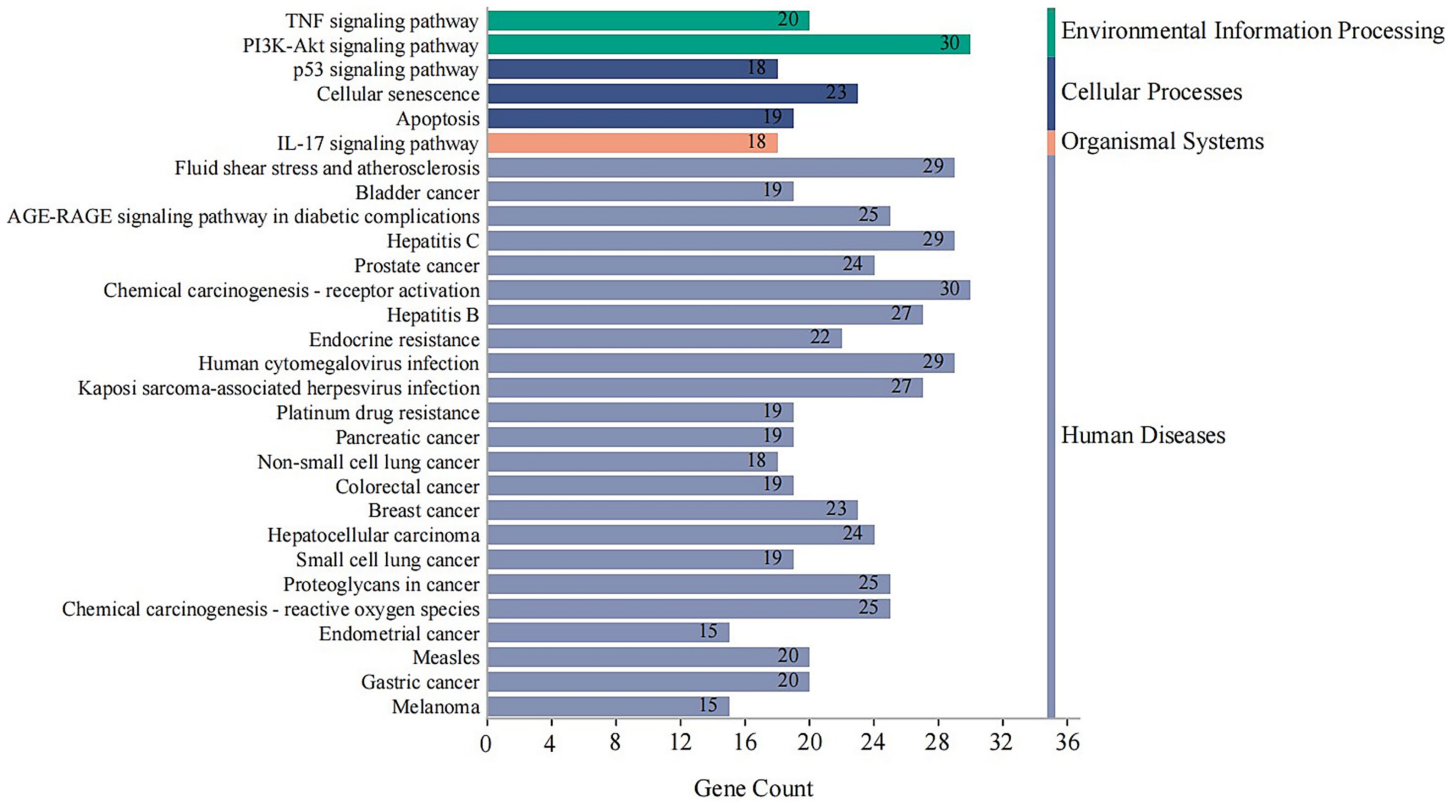
KEGG pathway enrichment analysis of core combination targets. This bar chart shows the top significantly enriched KEGG pathways for the 146 core targets. The x-axis represents the number of genes (Gene Count) enriched in each pathway, and the bars are colored according to their KEGG classification (e.g., Human Diseases, Cellular Processes). The analysis points to key pathways like TNF and PI3K-Akt signaling, which are central to inflammation, endocrine function, and cell survival. Enriched pathways with an adjusted p-value < 0.05 were considered significant.

KEGG Analysis: KEGG pathway enrichment analysis revealed that the 146 targets were significantly involved in pathways critical to human diseases and cellular processes. Key pathways included endocrine resistance, TNF signaling pathway, PI3K-Akt signaling pathway, apoptosis, and cellular senescence (Figure 8).

## 4 Discussion

Polycystic ovary syndrome (PCOS) is a multifactorial disorder influenced by a complex interplay of genetic, environmental, metabolic, and endocrine factors. A growing body of evidence highlights the critical role of inflammatory mechanisms in its pathogenesis, particularly oxidative stress and chronic low-grade inflammation (15). Elevated levels of inflammatory markers such as IL-6, IL-17, and TNF-*α* are commonly observed in PCOS patients (16, 17) and are closely associated with follicular developmental arrest, insulin resistance, and metabolic syndrome (18). This creates a vicious cycle that exacerbates the clinical manifestations of the disorder.

### 4.1 PCOS-infertility related targets

Our intersection analysis identified 2,500 shared targets between PCOS and infertility. From these, the TCMSP database and Cytoscape analysis helped pinpoint 40 core targets related to active herbal compounds. Among these, PTGS1 and PTGS2 are known to exacerbate chronic inflammation by promoting PGE2 synthesis, which stimulates androgen secretion from theca cells, thus aggravating insulin resistance and metabolic dysregulation (19). Sex hormone receptors (ESR1, ESR2, PGR, and AR) are increasingly recognized for their dual roles in both reproductive function and the modulation of inflammation and oxidative stress (20–23). Clinical evidence shows that DPP-4 inhibitors can reverse polycystic ovarian morphology and reduce serum androgen levels in women with PCOS (24). Additionally, dysfunction of the ADRB2 gene contributes to energy metabolism disturbances by impairing fatty acid release, and its polymorphisms have been linked to PCOS susceptibility (25). These identified core targets predominantly mediate processes of inflammation, oxidative stress, immune dysregulation, and metabolic dysfunction, aligning with the known pathophysiology of PCOS.

### 4.2 Prediction of PCOS-infertility related compounds

Among the identified active compounds, quercetin, kaempferol, and palmitic acid ranked highest by Degree value. Quercetin, a flavonoid with well-documented antioxidant, anti-inflammatory, and immunomodulatory properties, is widely investigated in gynecology (26). In PCOS models, it has been shown to suppress the Ox-LDL/TLR-4/NF-κB pathway, downregulating ovarian IL-1β, IL-6, and TNF-*α* expression (27), while also improving levels of SOD, CAT, and MDA to restore ovarian function (26). It also inhibits androgen receptor (AR) expression, thereby lowering androgen levels (28). Recent studies highlight its dual action on both ovarian tissue and the pituitary-ovarian axis to promote folliculogenesis, marking it as a promising therapeutic candidate for PCOS (29). Kaempferol, another flavonol, exhibits potent free radical scavenging and antioxidant effects, showing therapeutic potential for oxidative stress-related conditions (30). It has been found to reduce body weight, fasting glucose, and insulin resistance in PCOS rats by modulating hypothalamic inflammation and energy balance (31). Conversely, palmitic acid, a prevalent saturated fatty acid, is often elevated in the serum of PCOS patients (32) and is known to induce inflammation (33), oxidative stress (34), mitochondrial dysfunction, and insulin resistance *in vitro* (35, 36). The identification of these compounds aligns with the pathophysiology of PCOS, as they primarily target oxidative stress, chronic inflammation, and metabolic dysregulation.

### 4.3 Prediction of PCOS-infertility related herbs

The 488 herbs identified in this study were predominantly Warm and Pungent, followed by Cold, Bitter, Neutral, and Sweet medicines. This distribution reflects TCM’s therapeutic principles: Pungent-Warm herbs are used to resolve phlegm-dampness, Bitter-Cold herbs to clear heat-toxicity, and Sweet-Neutral herbs to tonify visceral functions. A clinical study of 234 PCOS patients revealed that the most prevalent TCM syndrome was spleen deficiency with phlegm-dampness (44.4%), followed by kidney deficiency with liver stagnation (36.3%) and phlegm-stasis interaction (12.0%) (37), which is consistent with our findings on herbal flavor distribution. The meridian tropism analysis emphasized the Lung, Liver, Stomach, Spleen, and Kidney meridians, aligning with therapeutic strategies to regulate qi, resolve phlegm, clear heat, and tonify organs. The top five herbs screened by Degree value (>20), namely *Ephedra sinica*, *Glycyrrhiza uralensis*, *Cinnamomum cassia*, *Bupleurum chinense*, and *Perilla frutescens*, may be considered priority candidates for treating PCOS-related infertility.

### 4.4 Core herbal combination for PCOS-related infertility

Through a comprehensive analysis of frequency statistics, association rules, and cluster analysis, we identified a core herbal combination: “*Ephedra sinica* (Mahuang)–*Magnolia officinalis* (Houpo)–*Bupleurum chinense* (Chaihu)–*Chrysanthemum morifolium* (Juhua)–*Angelica dahurica* (Baizhi)–*Morus alba* (Sangye).”

The rationale for this combination can be understood through TCM theory. Classical texts suggest a strong link between lung function and menstruation. For example, a passage in *The Yellow Emperor’s Inner Classic* implies that when lung qi is obstructed, its descending function is impaired, which can disrupt the connection to the uterus and lead to amenorrhea (38). Modern lifestyle factors, such as excessive consumption of cold and sweet foods, can damage yang qi and lead to internal phlegm-dampness. Sedentary habits and lack of exercise can further impair the diffusion of lung qi, causing phlegm-dampness obstruction, which blocks uterine collaterals and leads to menstrual disorders and infertility. This aligns with the TCM axiom, “The spleen is the source of phlegm production, while the lungs store the phlegm.” Therefore, regulating lung-spleen function may improve polycystic ovarian morphology in PCOS patients.

The core formula employs *Ephedra sinica*, *Bupleurum chinense*, and *Angelica dahurica* to diffuse lung qi and release the exterior; *Magnolia officinalis* to dry dampness and strengthen the spleen; and *Chrysanthemum morifolium* and *Morus alba* to clear heat and drain fire. Interestingly, the classical formula Wuji San, used for accumulations of “qi, blood, phlegm, fluid, and food,” contains *Ephedra sinica*, *Magnolia officinalis*, and *Angelica dahurica*. Research suggests Wuji San may regulate inflammatory factors, thereby improving ovulation and pregnancy rates in PCOS patients (39).

The compatibility of this formula can be analyzed from a TCM perspective. Ephedra acts as the monarch herb, powerfully diffusing lung qi to open blockages. It is assisted by Bupleurum, which soothes liver qi, and Angelica dahurica, which dispels wind-dampness, together addressing both the upper (lung) and middle (liver/spleen) jiao. Magnolia officinalis serves as the minister herb, drying dampness and promoting qi circulation to resolve the phlegm-dampness pathology. Chrysanthemum and *Morus alba* act as assistant and envoy herbs, clearing heat that may arise from stagnation and guiding the formula’s actions downward. From a safety perspective, the inclusion of heat-clearing herbs like Chrysanthemum and *Morus alba* helps to balance the warming and drying nature of Ephedra and Magnolia officinalis, reducing the risk of depleting yin or generating excess heat. However, the use of Ephedra requires caution due to its cardiovascular and central nervous system stimulant effects; its dosage and the patient’s condition must be carefully monitored in any future clinical application. This predicted synergy and balancing mechanism provides a strong rationale for its validation in animal models.

*Bupleurum chinense*, with its exterior-releasing and liver-soothing properties, is widely used for gynecological disorders, especially those related to emotional stress and liver qi stagnation (40, 41). For instance, Xiaoyao San, which features Bupleurum, may enhance endometrial receptivity in PCOS by regulating VEGF signaling (42). Similarly, historical texts note that *Morus alba* (mulberry leaf) and *Chrysanthemum morifolium* (chrysanthemum) are key herbs for treating uterine bleeding caused by liver heat (43), a condition that can manifest in PCOS. Modern TCM practitioners like Professor Shen Shaogong have successfully used modified versions of formulas containing these herbs to regulate yin-yang balance in PCOS patients (44).

Modern pharmacological studies lend further support to this combination. Ephedrine from *Ephedra sinica* has anti-inflammatory effects and may improve metabolic rate and circulation, countering insulin resistance (45). Magnolol from *Magnolia officinalis* demonstrates potent antioxidant and anti-inflammatory activity and improves insulin resistance (46–48). Active compounds in *Bupleurum chinense* modulate pathways like PI3K-AKT to improve glucose metabolism (49). Components of *Morus alba* (50), *Angelica dahurica* (51), and *Chrysanthemum morifolium* (52) have also been shown to improve glucose tolerance, insulin secretion, and lipid metabolism while fighting oxidative stress.

Our study’s findings are consistent with prior network pharmacology research on PCOS, yet offer a unique perspective due to the reverse methodology. For example, a study on the Guizhi Fulingwan formula identified targets related to inflammation and apoptosis, such as AKT1, TNF, and IL6 (53), which overlap with our findings. Another study on the Zishen Yutai Pill highlighted pathways in cancer and PI3K-Akt signaling (54). While these studies confirm the importance of inflammation and metabolic regulation, our reverse approach started from a broader disease-target landscape, leading to the identification of a novel herbal combination that emphasizes the role of the lung system in PCOS, a connection less explored in conventional network pharmacology analyses.

The GO enrichment analysis highlighted oxidative stress-related processes, while KEGG analysis pointed to endocrine resistance, TNF signaling, and PI3K-Akt signaling pathways. These findings collectively suggest that the core herbs treat PCOS-related infertility by ameliorating oxidative stress and endocrine dysfunction, which aligns perfectly with the functions of the identified PCOS-infertility targets.

### 4.5 Limitations

This study has several limitations that should be acknowledged. First, as a purely computational investigation, its findings are hypothesis-generating and lack experimental or clinical validation. Future studies involving cell culture experiments, animal models (e.g., letrozole-induced PCOS rats), and eventually clinical trials are necessary to verify the efficacy and safety of the predicted herbal combination. Second, the reliance on public databases for targets and compounds introduces potential biases, as some herbs or compounds may be underrepresented or incompletely annotated. The data does not account for tissue-specific gene expression or pharmacodynamics, which could influence therapeutic outcomes. Third, the statistical analysis of herbal properties, while informative, has inherent limitations. Our classification of herbs into therapeutic categories was based on their primary functions as documented in standardized texts, which does not account for their multiple effects or their specific roles (e.g., monarch, minister) within a formula. This could introduce bias, as suggested by the reviewer. For instance, high-frequency herbs with broad applications, such as licorice, might influence the distribution across categories. Future studies could employ weighted statistical methods and incorporate multi-center prescription data to refine these findings. The suggestion to use a Sankey diagram to visualize channel-efficacy flow is excellent and represents a valuable direction for future, more granular analyses. Fourth, the screening criteria for active compounds (e.g., high GI absorption, druglikeness) might have inadvertently excluded potentially effective natural metabolites that do not meet these stringent pharmaceutical filters but could still exert biological effects. Fifth, this study did not address crucial clinical considerations such as optimal dosage, potential herb-drug interactions, or toxicity of the proposed herbs. Finally, PCOS is a highly heterogeneous syndrome with multiple phenotypes (e.g., lean vs. obese PCOS) and diverse TCM syndromic patterns. Our analysis treated PCOS as a single entity and did not differentiate between these subtypes, which may require tailored therapeutic approaches.

## 5 Conclusion

This reverse network pharmacology study identified 2,500 shared targets and 1,545 active compounds relevant to PCOS-related infertility, culminating in the prediction of a core herbal combination: “*Ephedra sinica*–*Magnolia officinalis*–*Bupleurum chinense*–*Chrysanthemum morifolium*–*Angelica dahurica*–*Morus alba*.” These herbs are predicted to synergistically target oxidative stress, inflammation, and endocrine dysregulation. While further experimental validation is essential, these findings offer a novel integrative strategy for PCOS management and provide a theoretical foundation for future clinical practice and mechanistic research.

## Data Availability

The original contributions presented in the study are included in the article/Supplementary material, further inquiries can be directed to the corresponding authors.

## Footnotes

^1^ https://www.genecards.org

^2^ https://www.omim.org

^3^ https://www.pharmgkb.org

^4^ https://disgenet.com

^5^ https://go.drugbank.com

^6^ https://db.idrblab.net/ttd

^7^ https://old.tcmsp-e.com/

^8^ https://pubchem.ncbi.nlm.nih.gov/

^9^ http://www.swissadme.ch/
