# Supplementary material for: A reverse network pharmacology and bioinformatics-based approach to exploring medication patterns for polycystic ovary syndrome-related infertility
Source: Front Med (Lausanne). 2025 Nov 5;12:1614165. doi: 10.3389/fmed.2025.1614165 (PMC12626921; doi:10.3389/fmed.2025.1614165)
Supplement: Supplementary file 1 [file Table_1.DOCX]

**Supplementary Table S1. Full List of 1,545 Active Compounds Associated with PCOS-Related Infertility**

| **No.** | **Chemical Name** | **Mol ID** | **PubChem ID** | **GI Absorption** | **Number of Druglikeness "Yes" Criteria** |
| --- | --- | --- | --- | --- | --- |
| 1 | Quercetin | MOL000098 | 5280343 | High | 5 |
| 2 | Kaempferol | MOL000422 | 5280863 | High | 5 |
| 3 | 17-beta-estradiol | MOL010919 | 5757 | High | 5 |
| 4 | (S)-Scoulerine | MOL000217 | 439654 | High | 5 |
| 5 | L-Bornyl acetate | MOL000196 | 93009 | High | 4 |
| 6 | Azeton | MOL004472 | 180 | High | 3 |
| 7 | IPA | MOL008719 | 3776 | High | 3 |
| 8 | (L)-alpha-Terpineol | MOL000118 | 443162 | High | 3 |
| 9 | (R)-linalool | MOL000198 | 443158 | High | 3 |
| 10 | (2R)-2-methylbutan-1-ol | MOL009813 | 637572 | High | 3 |
| 11 | Propionic ether | MOL009843 | 7749 | High | 3 |
| 12 | 2-methylbut-2-en-1-ol | MOL010596 | 6433417 | High | 3 |
| 13 | ZINC02037591 | MOL010665 | 6999854 | High | 3 |
| 14 | 3-cyanopropanamide | MOL012808 | 3017295 | High | 3 |
| 15 | Methyl butyrate | MOL013372 | 12180 | High | 3 |
| 16 | Palmitic acid | MOL000069 | 985 | High | 2 |
| 17 | Luteolin | MOL000006 | 5280445 | High | 4 |
| 18 | beta-Sitosterol | MOL000359 | 222284 | High | 3 |
| 19 | Stigmasterol | MOL000452 | 5280794 | High | 3 |
| 20 | Apigenin | MOL000010 | 5280443 | High | 4 |
| 21 | Naringenin | MOL000173 | 932 | High | 5 |
| 22 | Isorhamnetin | MOL000358 | 5281654 | High | 4 |
| 23 | 7-Methoxy-2-methyl isoflavone | MOL001715 | 5282121 | High | 3 |
| 24 | Formononetin | MOL001716 | 5280378 | High | 3 |
| 25 | beta-carotene | MOL000548 | 5280489 | High | 2 |
| 26 | Baicalein | MOL002428 | 5281605 | High | 3 |
| 27 | Wogonin | MOL001729 | 5281635 | High | 3 |
| 28 | hespiridin | MOL000004 | 10621 | High | 2 |
| 29 | Nobiletin | MOL000388 | 72363 | High | 3 |
| 30 | Tangeretin | MOL000412 | 72364 | High | 3 |
| 31 | Curcumin | MOL000212 | 969516 | High | 3 |
| 32 | Berberine | MOL001725 | 2353 | High | 5 |
| 33 | Cinnamic acid | MOL000350 | 444539 | High | 3 |
| 34 | Gallic acid | MOL000168 | 370 | High | 4 |
| 35 | Camphor | MOL000215 | 2537 | High | 3 |
| 36 | Menthol | MOL000191 | 1254 | High | 3 |
| 37 | Eugenol | MOL000222 | 3314 | High | 3 |
| 38 | Thymol | MOL000216 | 6989 | High | 3 |
| 39 | Carvacrol | MOL000214 | 10364 | High | 3 |
| 40 | Anethole | MOL007425 | 637563 | High | 3 |
| 41 | Limonene | MOL000473 | 22311 | High | 4 |
| 42 | alpha-Pinene | MOL000523 | 6654 | High | 4 |
| 43 | beta-Pinene | MOL001460 | 14896 | High | 4 |
| 44 | 1,8-Cineole | MOL000407 | 2758 | High | 3 |
| 45 | Sabinene | MOL001550 | 18818 | High | 4 |
| 46 | gamma-Terpinene | MOL001584 | 7461 | High | 4 |
| 47 | p-Cymene | MOL000401 | 7463 | High | 3 |
| 48 | Myrcene | MOL000474 | 31253 | High | 4 |
| 49 | beta-Caryophyllene | MOL000494 | 5281515 | High | 5 |
| 50 | alpha-Humulene | MOL000511 | 5281520 | High | 4 |
| 51 | Geraniol | MOL000301 | 637566 | High | 3 |
| 52 | Nerol | MOL000302 | 637590 | High | 3 |
| 53 | Citronellal | MOL000287 | 7794 | High | 3 |
| 54 | Citral | MOL000286 | 638011 | High | 3 |
| 55 | Eucalyptol | MOL000468 | 2758 | High | 3 |
| 56 | Fenchone | MOL004123 | 8453 | High | 3 |
| 57 | alpha-Thujone | MOL001525 | 11030 | High | 3 |
| 58 | beta-Thujone | MOL001526 | 62419 | High | 3 |
| 59 | Pulegone | MOL001543 | 6986 | High | 3 |
| 60 | Magnolol | MOL002441 | 72276 | High | 3 |
| 61 | Honokiol | MOL002442 | 72277 | High | 3 |
| 62 | Saikosaponin A | MOL004116 | 107736 | High | 2 |
| 63 | Saikosaponin D | MOL004119 | 119053 | High | 2 |
| 64 | Genistein | MOL000350 | 5280961 | High | 4 |
| 65 | Daidzein | MOL000349 | 5280379 | High | 4 |
| 66 | Glycitein | MOL001719 | 5317750 | High | 4 |
| 67 | Puerarin | MOL000078 | 5281812 | High | 3 |
| 68 | Salidroside | MOL000392 | 145850 | High | 3 |
| 69 | Rosmarinic acid | MOL000251 | 5281792 | High | 3 |
| 70 | Ursolic acid | MOL000109 | 64945 | High | 2 |
| 71 | Oleanolic acid | MOL000391 | 10494 | High | 2 |
| 72 | Betulinic acid | MOL000110 | 64971 | High | 2 |
| 73 | Hederagenin | MOL003323 | 10156 | High | 2 |
| 74 | Ginsenoside Rb1 | MOL005321 | 6325996 | High | 2 |
| 75 | Ginsenoside Rg1 | MOL005322 | 5484859 | High | 2 |
| 76 | Caffeic acid | MOL000348 | 689043 | High | 4 |
| 77 | Ferulic acid | MOL000354 | 445858 | High | 4 |
| 78 | Sinapic acid | MOL000410 | 637775 | High | 4 |
| 79 | Chlorogenic acid | MOL000347 | 1794427 | High | 3 |
| 80 | Catechin | MOL000234 | 9064 | High | 3 |
| 81 | Epicatechin | MOL000235 | 72276 | High | 3 |
| 82 | Gallocatechin | MOL002431 | 65084 | High | 3 |
| 83 | Epigallocatechin | MOL002432 | 9883506 | High | 3 |
| 84 | EGCG | MOL000219 | 65064 | High | 2 |
| 85 | Theaflavin | MOL002433 | 114777 | High | 2 |
| 86 | Resveratrol | MOL000424 | 445154 | High | 3 |
| 87 | Piceatannol | MOL004100 | 667638 | High | 3 |
| 88 | Pterostilbene | MOL001726 | 5281783 | High | 3 |
| 89 | Piperine | MOL000593 | 638024 | High | 3 |
| 90 | Capsaicin | MOL000346 | 1548943 | High | 4 |
| 91 | Gingerol | MOL000164 | 9997 | High | 3 |
| 92 | Shogaol | MOL007460 | 10044 | High | 3 |
| 93 | Zingerone | MOL007461 | 31211 | High | 3 |
| 94 | Aloin | MOL002404 | 10189 | High | 2 |
| 95 | Emodin | MOL000220 | 3220 | High | 3 |
| 96 | Rhein | MOL000221 | 10129 | High | 3 |
| 97 | Sennoside A | MOL000406 | 73254 | High | 2 |
| 98 | Sennoside B | MOL000407 | 442340 | High | 2 |
| 99 | Astragaloside IV | MOL002931 | 13943433 | High | 2 |
| 100 | Rutin | MOL000088 | 5280805 | High | 2 |
| 101 | Glycine | MOL795279 | 3580651 | High | 3 |
| 102 | Arachidonic acid | MOL864277 | 2794413 | High | 2 |
| 103 | Zearalenone | MOL223303 | 5724269 | High | 4 |
| 104 | Psoralen derivative | MOL145368 | 9360704 | High | 5 |
| 105 | Tryptophan | MOL839849 | 14271236 | High | 2 |
| 106 | Artemisinin | MOL946558 | 10008502 | High | 4 |
| 107 | scopoletin | MOL181632 | 1282160 | High | 5 |
| 108 | Docosahexaenoic acid (DHA) | MOL147770 | 4997499 | High | 2 |
| 109 | Matrine | MOL476093 | 5280085 | High | 3 |
| 110 | Carnosic acid | MOL118576 | 10330450 | High | 4 |
| 111 | aesculetin derivative | MOL226648 | 1931825 | High | 4 |
| 112 | Xanthohumol | MOL717497 | 2138345 | High | 5 |
| 113 | aesculetin | MOL235220 | 2200264 | High | 3 |
| 114 | Diosgenin | MOL563441 | 10019472 | High | 5 |
| 115 | Isoleucine | MOL678818 | 10587308 | High | 4 |
| 116 | Tyrosine | MOL693101 | 10470729 | High | 2 |
| 117 | Geranyl acetate derivative | MOL448433 | 6637560 | High | 2 |
| 118 | Diosgenin derivative | MOL562308 | 6110394 | High | 5 |
| 119 | Arachidonic acid 119 | MOL295668 | 8868166 | High | 4 |
| 120 | Threonine derivative | MOL488252 | 3785055 | High | 4 |
| 121 | coumarin derivative | MOL181995 | 7196752 | High | 5 |
| 122 | Icariin derivative | MOL877707 | 4448417 | High | 2 |
| 123 | Silymarin | MOL791540 | 8617966 | High | 4 |
| 124 | Yohimbine | MOL177194 | 1985173 | High | 4 |
| 125 | Ellagic acid | MOL872400 | 11599251 | High | 3 |
| 126 | Isoleucine 126 | MOL871197 | 12341238 | High | 3 |
| 127 | Glutathione | MOL371471 | 4866505 | High | 4 |
| 128 | Farnesol | MOL758244 | 1175602 | High | 3 |
| 129 | Ellagic acid 129 | MOL372141 | 5304205 | High | 4 |
| 130 | Eicosapentaenoic acid (EPA) derivative | MOL563030 | 1354356 | High | 4 |
| 131 | coumarin derivative 131 | MOL130188 | 14714554 | High | 5 |
| 132 | Isoleucine 132 | MOL381119 | 3550167 | High | 4 |
| 133 | Alpha-Linolenic acid | MOL188681 | 9756646 | High | 3 |
| 134 | Icariin | MOL918270 | 10400686 | High | 4 |
| 135 | Glutathione 135 | MOL384333 | 3293238 | High | 5 |
| 136 | Borneol | MOL378353 | 1099520 | High | 5 |
| 137 | Gamma-Linolenic acid | MOL147666 | 10972400 | High | 2 |
| 138 | Hesperetin | MOL714375 | 12523039 | High | 5 |
| 139 | Docosahexaenoic acid (DHA) 139 | MOL458148 | 14499277 | High | 5 |
| 140 | Ergothioneine | MOL383198 | 2053957 | High | 4 |
| 141 | Serine | MOL306207 | 13393971 | High | 5 |
| 142 | Myristic acid derivative | MOL967357 | 8430203 | High | 4 |
| 143 | capric acid | MOL473864 | 3512357 | High | 4 |
| 144 | Citronellol | MOL649199 | 14015186 | High | 5 |
| 145 | Cedrol | MOL862485 | 12632214 | High | 2 |
| 146 | Verbenone | MOL844221 | 1991775 | High | 4 |
| 147 | Stevioside | MOL177513 | 11154400 | High | 4 |
| 148 | aesculetin 148 | MOL305848 | 5731600 | High | 3 |
| 149 | Saponin | MOL313983 | 9349631 | High | 3 |
| 150 | Tryptophan 150 | MOL770545 | 6965879 | High | 5 |
| 151 | Tryptophan 151 | MOL984305 | 14551156 | High | 2 |
| 152 | Ellagic acid derivative | MOL933402 | 951544 | High | 4 |
| 153 | Galangin | MOL439508 | 14051059 | High | 2 |
| 154 | Taurine | MOL656134 | 8852444 | High | 3 |
| 155 | Parthenolide | MOL637478 | 12278710 | High | 4 |
| 156 | Stevioside 156 | MOL656453 | 13367571 | High | 5 |
| 157 | Linalyl acetate | MOL656479 | 9147209 | High | 2 |
| 158 | aesculetin 158 | MOL701381 | 13736005 | High | 5 |
| 159 | Nerolidol | MOL891072 | 1418869 | High | 4 |
| 160 | Tryptophan 160 | MOL197936 | 10254390 | High | 3 |
| 161 | Farnesol derivative | MOL820317 | 5713327 | High | 3 |
| 162 | Stevioside 162 | MOL217916 | 6210845 | High | 2 |
| 163 | Saponin 163 | MOL831708 | 7195165 | High | 2 |
| 164 | Choline | MOL586363 | 7308041 | High | 3 |
| 165 | coumarin | MOL103832 | 4244742 | High | 3 |
| 166 | Procyanidin B2 | MOL469372 | 10399376 | High | 3 |
| 167 | Myristic acid | MOL510882 | 2062682 | High | 2 |
| 168 | Betaine | MOL654493 | 6742077 | High | 3 |
| 169 | Colchicine | MOL430740 | 10818226 | High | 5 |
| 170 | Citronellol 170 | MOL783138 | 9839630 | High | 4 |
| 171 | Inositol | MOL473770 | 7247733 | High | 5 |
| 172 | Proline | MOL811028 | 3322409 | High | 4 |
| 173 | Linalyl acetate derivative | MOL866879 | 3838811 | High | 4 |
| 174 | Osthole | MOL562509 | 6363191 | High | 3 |
| 175 | Betaine 175 | MOL533206 | 5385400 | High | 3 |
| 176 | Myristic acid derivative 176 | MOL254190 | 2476433 | High | 3 |
| 177 | Choline derivative | MOL920747 | 6417441 | High | 5 |
| 178 | Isoeugenol | MOL815896 | 6533782 | High | 4 |
| 179 | Patchouli alcohol | MOL518616 | 9684923 | High | 4 |
| 180 | Phloretin | MOL755616 | 3576228 | High | 5 |
| 181 | Ergothioneine 181 | MOL367218 | 11672528 | High | 2 |
| 182 | Sinomenine | MOL847445 | 9240981 | High | 5 |
| 183 | Choline 183 | MOL477121 | 398078 | High | 3 |
| 184 | Myricetin | MOL549825 | 12055392 | High | 3 |
| 185 | Fisetin derivative | MOL372546 | 647078 | High | 4 |
| 186 | Isoeugenol 186 | MOL296895 | 11003652 | High | 2 |
| 187 | Echinacoside | MOL625257 | 14263385 | High | 5 |
| 188 | Andrographolide | MOL846809 | 4533410 | High | 4 |
| 189 | Baicalin derivative | MOL551856 | 1715852 | High | 5 |
| 190 | Alpha-Bisabolol | MOL525990 | 12439457 | High | 2 |
| 191 | butyric acid derivative | MOL442471 | 4862569 | High | 4 |
| 192 | Stevioside 192 | MOL720820 | 5323987 | High | 3 |
| 193 | Tryptophan 193 | MOL981442 | 14801445 | High | 2 |
| 194 | Sinomenine 194 | MOL586541 | 13437019 | High | 2 |
| 195 | Isoeugenol 195 | MOL790944 | 7383359 | High | 4 |
| 196 | Alanine | MOL473190 | 6922234 | High | 3 |
| 197 | Docosahexaenoic acid (DHA) 197 | MOL258147 | 13033416 | High | 2 |
| 198 | Oleic acid | MOL763360 | 6461164 | High | 5 |
| 199 | Baicalin derivative 199 | MOL627652 | 11283620 | High | 2 |
| 200 | Arachidonic acid 200 | MOL589782 | 5070345 | High | 2 |
| 201 | Oleic acid 201 | MOL167141 | 9574377 | High | 5 |
| 202 | Creatine | MOL690853 | 1624138 | High | 2 |
| 203 | Cedrol 203 | MOL127071 | 14862156 | High | 4 |
| 204 | Saponin 204 | MOL601137 | 9254773 | High | 3 |
| 205 | Artemisinin derivative | MOL792493 | 2504629 | High | 4 |
| 206 | Arginine | MOL830666 | 504501 | High | 3 |
| 207 | vanillin | MOL872269 | 5497078 | High | 2 |
| 208 | Patchouli alcohol derivative | MOL896409 | 7887650 | High | 2 |
| 209 | Echinacoside 209 | MOL101369 | 13393347 | High | 5 |
| 210 | capric acid 210 | MOL210699 | 11407059 | High | 3 |
| 211 | Nerolidol 211 | MOL311061 | 6647161 | High | 3 |
| 212 | Xanthohumol 212 | MOL183168 | 5843823 | High | 4 |
| 213 | Myricetin derivative | MOL325888 | 3919217 | High | 2 |
| 214 | Terpinen-4-ol | MOL450505 | 11784960 | High | 4 |
| 215 | Glutamic acid | MOL134877 | 14008934 | High | 4 |
| 216 | aesculetin 216 | MOL212246 | 9655530 | High | 4 |
| 217 | Verbenone derivative | MOL977469 | 6656791 | High | 2 |
| 218 | Valine | MOL612863 | 2286764 | High | 4 |
| 219 | Parthenolide 219 | MOL417791 | 5035006 | High | 5 |
| 220 | Patchouli alcohol 220 | MOL653270 | 13619411 | High | 5 |
| 221 | Colchicine 221 | MOL654471 | 5351781 | High | 5 |
| 222 | Salicin | MOL884763 | 6768281 | High | 4 |
| 223 | Glycine 223 | MOL701120 | 2934968 | High | 4 |
| 224 | Carnosic acid 224 | MOL424728 | 3841365 | High | 2 |
| 225 | Tyrosine 225 | MOL706487 | 257685 | High | 4 |
| 226 | Taxifolin derivative | MOL353584 | 11738638 | High | 3 |
| 227 | Baicalin derivative 227 | MOL695240 | 2649249 | High | 3 |
| 228 | Patchouli alcohol 228 | MOL267012 | 12106816 | High | 4 |
| 229 | Sinomenine 229 | MOL901809 | 893652 | High | 4 |
| 230 | Linoleic acid | MOL808785 | 10614000 | High | 5 |
| 231 | Glutamine derivative | MOL287947 | 1334064 | High | 5 |
| 232 | Myristic acid 232 | MOL176911 | 12920852 | High | 2 |
| 233 | Lecithin | MOL430718 | 12225742 | High | 5 |
| 234 | Xanthohumol derivative | MOL913244 | 6867155 | High | 4 |
| 235 | ethyl vanillin | MOL950245 | 1843088 | High | 5 |
| 236 | Threonine | MOL470371 | 1518030 | High | 3 |
| 237 | Tyrosine derivative | MOL242071 | 3439001 | High | 2 |
| 238 | Betaine 238 | MOL479954 | 14238316 | High | 2 |
| 239 | Osthole 239 | MOL963831 | 2086736 | High | 4 |
| 240 | Echinacoside derivative | MOL753729 | 14015440 | High | 5 |
| 241 | Isoeugenol derivative | MOL825575 | 13611738 | High | 4 |
| 242 | Zearalenone derivative | MOL103563 | 9080542 | High | 2 |
| 243 | Icariin 243 | MOL847794 | 12918896 | High | 5 |
| 244 | coumarin 244 | MOL412564 | 13396987 | High | 4 |
| 245 | Verbenone 245 | MOL565959 | 7147398 | High | 5 |
| 246 | Scutellarin | MOL254585 | 5075851 | High | 5 |
| 247 | Citronellol 247 | MOL149668 | 8564562 | High | 2 |
| 248 | Chrysin | MOL990696 | 3435166 | High | 5 |
| 249 | Yohimbine 249 | MOL558409 | 5623566 | High | 4 |
| 250 | Andrographolide 250 | MOL111405 | 1055666 | High | 2 |
| 251 | Yohimbine derivative | MOL547678 | 5451611 | High | 5 |
| 252 | Inositol 252 | MOL638201 | 11419966 | High | 3 |
| 253 | Triptolide | MOL430850 | 1584364 | High | 3 |
| 254 | Proline 254 | MOL815535 | 1805280 | High | 5 |
| 255 | Colchicine 255 | MOL234013 | 9045573 | High | 4 |
| 256 | Serine 256 | MOL447390 | 7868965 | High | 3 |
| 257 | Asparagine | MOL488999 | 514981 | High | 4 |
| 258 | Alpha-Bisabolol 258 | MOL577352 | 2393448 | High | 4 |
| 259 | Carnosic acid 259 | MOL177149 | 14453419 | High | 4 |
| 260 | Myristic acid 260 | MOL183956 | 7742771 | High | 3 |
| 261 | syringaldehyde | MOL258391 | 9645107 | High | 5 |
| 262 | Gamma-Linolenic acid 262 | MOL295180 | 9614914 | High | 2 |
| 263 | Glycine 263 | MOL838522 | 3127192 | High | 4 |
| 264 | Zearalenone 264 | MOL157003 | 4225155 | High | 3 |
| 265 | Plumbagin | MOL730077 | 13610433 | High | 3 |
| 266 | Taurine 266 | MOL335132 | 6483492 | High | 3 |
| 267 | Procyanidin B2 267 | MOL585833 | 15075213 | High | 2 |
| 268 | Glutathione 268 | MOL488841 | 5997973 | High | 5 |
| 269 | Borneol 269 | MOL719973 | 3921782 | High | 3 |
| 270 | Baicalin derivative 270 | MOL712440 | 1173000 | High | 2 |
| 271 | Eicosapentaenoic acid (EPA) | MOL415685 | 8486483 | High | 4 |
| 272 | Myristic acid 272 | MOL990054 | 12057984 | High | 3 |
| 273 | Farnesol derivative 273 | MOL155488 | 5587115 | High | 2 |
| 274 | Betaine 274 | MOL174827 | 9448190 | High | 3 |
| 275 | Oleic acid 275 | MOL802365 | 14869787 | High | 3 |
| 276 | Psoralen | MOL342802 | 3051356 | High | 3 |
| 277 | Taxifolin derivative 277 | MOL796869 | 10363473 | High | 4 |
| 278 | Isoeugenol 278 | MOL960455 | 3232204 | High | 3 |
| 279 | capric acid 279 | MOL939897 | 2706745 | High | 3 |
| 280 | Linoleic acid derivative | MOL400020 | 12153584 | High | 5 |
| 281 | Vincristine | MOL987611 | 6659282 | High | 5 |
| 282 | Taxifolin | MOL992048 | 2577261 | High | 4 |
| 283 | Vincristine 283 | MOL643326 | 4090546 | High | 2 |
| 284 | Osthole 284 | MOL890979 | 12363803 | High | 2 |
| 285 | capric acid 285 | MOL377879 | 9612517 | High | 5 |
| 286 | Carnosic acid derivative | MOL916127 | 7897794 | High | 3 |
| 287 | caprylic acid | MOL418903 | 14498707 | High | 5 |
| 288 | Silymarin 288 | MOL149407 | 10608145 | High | 3 |
| 289 | Betaine 289 | MOL413226 | 8686969 | High | 4 |
| 290 | Psoralen 290 | MOL773980 | 3858177 | High | 4 |
| 291 | Glycine 291 | MOL412362 | 628645 | High | 2 |
| 292 | Celastrol | MOL513252 | 8878065 | High | 2 |
| 293 | Glutathione 293 | MOL111460 | 1010174 | High | 4 |
| 294 | Threonine 294 | MOL254090 | 7408095 | High | 3 |
| 295 | Docosahexaenoic acid (DHA) 295 | MOL384163 | 7373246 | High | 3 |
| 296 | Diosgenin 296 | MOL487207 | 13207527 | High | 5 |
| 297 | Glutathione 297 | MOL753195 | 11804071 | High | 3 |
| 298 | Isoeugenol 298 | MOL403909 | 3493615 | High | 3 |
| 299 | Myricetin 299 | MOL958825 | 3480541 | High | 4 |
| 300 | Psoralen derivative 300 | MOL231812 | 4089867 | High | 3 |
| 301 | Hesperetin 301 | MOL762728 | 13282903 | High | 4 |
| 302 | Plumbagin 302 | MOL971877 | 6193563 | High | 2 |
| 303 | Oleic acid 303 | MOL881368 | 8406694 | High | 4 |
| 304 | Carnosic acid derivative 304 | MOL808940 | 12025610 | High | 5 |
| 305 | Choline 305 | MOL419364 | 14938382 | High | 4 |
| 306 | scopoletin 306 | MOL428081 | 14098288 | High | 4 |
| 307 | Procyanidin B2 307 | MOL619331 | 8451875 | High | 3 |
| 308 | Terpinen-4-ol 308 | MOL483784 | 2184678 | High | 2 |
| 309 | Glutamic acid 309 | MOL888918 | 178258 | High | 2 |
| 310 | Alpha-Bisabolol 310 | MOL373315 | 2037336 | High | 3 |
| 311 | Isoeugenol 311 | MOL349595 | 14517438 | High | 4 |
| 312 | capric acid 312 | MOL825139 | 13810455 | High | 5 |
| 313 | Verbenone 313 | MOL896576 | 12653773 | High | 3 |
| 314 | Myricetin derivative 314 | MOL396462 | 13055749 | High | 2 |
| 315 | Zearalenone 315 | MOL781724 | 7559067 | High | 5 |
| 316 | Glutamic acid 316 | MOL634812 | 14590169 | High | 3 |
| 317 | caprylic acid 317 | MOL756797 | 13176988 | High | 3 |
| 318 | caproic acid | MOL567220 | 7353866 | High | 3 |
| 319 | Xanthohumol 319 | MOL770003 | 8126793 | High | 5 |
| 320 | Leucine | MOL609281 | 13965353 | High | 5 |
| 321 | Andrographolide 321 | MOL261264 | 1634985 | High | 3 |
| 322 | Scutellarin 322 | MOL394515 | 10739937 | High | 2 |
| 323 | Glutamine | MOL504065 | 12022901 | High | 2 |
| 324 | Echinacoside 324 | MOL421560 | 11409296 | High | 2 |
| 325 | Yohimbine 325 | MOL540987 | 6230009 | High | 2 |
| 326 | Glycine 326 | MOL974164 | 116179 | High | 5 |
| 327 | Linalyl acetate 327 | MOL970195 | 12143898 | High | 3 |
| 328 | Celastrol 328 | MOL174788 | 4977434 | High | 2 |
| 329 | Linalyl acetate derivative 329 | MOL475799 | 5173512 | High | 3 |
| 330 | Myricetin 330 | MOL819336 | 13557705 | High | 3 |
| 331 | Valine 331 | MOL888221 | 2756892 | High | 2 |
| 332 | Colchicine 332 | MOL951594 | 5302493 | High | 5 |
| 333 | capric acid derivative | MOL606378 | 4242882 | High | 3 |
| 334 | Glutamine 334 | MOL235489 | 9700464 | High | 4 |
| 335 | Linoleic acid derivative 335 | MOL119805 | 3935792 | High | 4 |
| 336 | Stevioside 336 | MOL522286 | 14202166 | High | 5 |
| 337 | Nerolidol derivative | MOL236214 | 6258726 | High | 4 |
| 338 | Creatine 338 | MOL250464 | 8263003 | High | 3 |
| 339 | Docosahexaenoic acid (DHA) 339 | MOL568823 | 12214147 | High | 2 |
| 340 | Diosgenin 340 | MOL749364 | 3170346 | High | 2 |
| 341 | Linalyl acetate 341 | MOL863771 | 5252022 | High | 2 |
| 342 | Myricetin 342 | MOL221022 | 7221366 | High | 4 |
| 343 | Cedrol 343 | MOL367325 | 14607168 | High | 4 |
| 344 | Glutathione 344 | MOL183633 | 1167913 | High | 4 |
| 345 | Alanine derivative | MOL907048 | 7585143 | High | 4 |
| 346 | Vincristine 346 | MOL964556 | 6237886 | High | 4 |
| 347 | Phloretin 347 | MOL907277 | 7783168 | High | 2 |
| 348 | Proline 348 | MOL675035 | 3261986 | High | 5 |
| 349 | Phloretin derivative | MOL941596 | 4143266 | High | 4 |
| 350 | capric acid derivative 350 | MOL114227 | 11326262 | High | 4 |
| 351 | Diosgenin 351 | MOL692785 | 11591895 | High | 2 |
| 352 | Withaferin A | MOL232053 | 4788220 | High | 2 |
| 353 | aesculetin derivative 353 | MOL733115 | 13247683 | High | 4 |
| 354 | Stearic acid | MOL221345 | 9712869 | High | 4 |
| 355 | capric acid 355 | MOL257713 | 12070067 | High | 4 |
| 356 | scopoletin derivative | MOL489066 | 2215749 | High | 2 |
| 357 | Withaferin A 357 | MOL890218 | 2040826 | High | 3 |
| 358 | Ellagic acid 358 | MOL675674 | 13826426 | High | 5 |
| 359 | Farnesol 359 | MOL624791 | 14262807 | High | 2 |
| 360 | Silymarin 360 | MOL787821 | 7388045 | High | 3 |
| 361 | Ellagic acid 361 | MOL837585 | 4288095 | High | 3 |
| 362 | Oleic acid 362 | MOL491371 | 6588492 | High | 3 |
| 363 | Saponin 363 | MOL218656 | 8080955 | High | 4 |
| 364 | Myricetin derivative 364 | MOL335480 | 4260429 | High | 2 |
| 365 | Icariin 365 | MOL145910 | 3244828 | High | 3 |
| 366 | Silymarin 366 | MOL844053 | 13071953 | High | 2 |
| 367 | Triptolide 367 | MOL677473 | 7348199 | High | 4 |
| 368 | Eicosapentaenoic acid (EPA) derivative 368 | MOL128172 | 12446455 | High | 5 |
| 369 | Icariin 369 | MOL899296 | 4359888 | High | 2 |
| 370 | Terpinen-4-ol derivative | MOL854614 | 11833641 | High | 4 |
| 371 | Saponin 371 | MOL196213 | 6107726 | High | 4 |
| 372 | Stevioside 372 | MOL942231 | 14622492 | High | 4 |
| 373 | Stearic acid 373 | MOL915291 | 472302 | High | 2 |
| 374 | Geranyl acetate derivative 374 | MOL715924 | 5964196 | High | 5 |
| 375 | Vincristine 375 | MOL913527 | 8214555 | High | 2 |
| 376 | Celastrol 376 | MOL814826 | 2335370 | High | 2 |
| 377 | coumarin 377 | MOL871181 | 4416418 | High | 2 |
| 378 | Alpha-Linolenic acid 378 | MOL527947 | 12403569 | High | 5 |
| 379 | vanillin derivative | MOL968416 | 11278972 | High | 4 |
| 380 | Saponin derivative | MOL871732 | 6885744 | High | 2 |
| 381 | Gamma-Linolenic acid 381 | MOL772605 | 491096 | High | 4 |
| 382 | syringaldehyde 382 | MOL412697 | 14010313 | High | 4 |
| 383 | Colchicine derivative | MOL727537 | 2064540 | High | 3 |
| 384 | Galangin 384 | MOL252279 | 11194776 | High | 4 |
| 385 | Creatine 385 | MOL117234 | 2809006 | High | 4 |
| 386 | Alanine 386 | MOL429075 | 12345220 | High | 2 |
| 387 | Isoeugenol 387 | MOL383682 | 14211223 | High | 3 |
| 388 | Farnesol 388 | MOL922441 | 6200158 | High | 4 |
| 389 | Serine derivative | MOL140270 | 10392393 | High | 3 |
| 390 | Baicalin derivative 390 | MOL320293 | 14522392 | High | 5 |
| 391 | Gamma-Linolenic acid 391 | MOL906929 | 1828917 | High | 5 |
| 392 | Terpinen-4-ol 392 | MOL263273 | 5129469 | High | 3 |
| 393 | Nerolidol derivative 393 | MOL880969 | 2079922 | High | 4 |
| 394 | Icariin 394 | MOL951589 | 13318594 | High | 4 |
| 395 | Patchouli alcohol 395 | MOL690637 | 1476424 | High | 4 |
| 396 | Stevioside derivative | MOL171750 | 3181698 | High | 4 |
| 397 | Inositol 397 | MOL703888 | 14990587 | High | 2 |
| 398 | Saponin derivative 398 | MOL342353 | 12498730 | High | 2 |
| 399 | Xanthohumol 399 | MOL650683 | 3615403 | High | 5 |
| 400 | Terpinen-4-ol 400 | MOL468402 | 12187701 | High | 5 |
| 401 | Arachidonic acid derivative | MOL978825 | 13959835 | High | 5 |
| 402 | Silymarin 402 | MOL348153 | 5850135 | High | 2 |
| 403 | Creatine 403 | MOL293732 | 10186326 | High | 4 |
| 404 | Farnesol 404 | MOL498477 | 3862100 | High | 3 |
| 405 | Choline 405 | MOL506809 | 8531505 | High | 3 |
| 406 | Glutamic acid 406 | MOL549032 | 12391070 | High | 5 |
| 407 | Echinacoside 407 | MOL302301 | 112826 | High | 4 |
| 408 | Creatine 408 | MOL118361 | 3879413 | High | 3 |
| 409 | Proline 409 | MOL394298 | 374460 | High | 4 |
| 410 | Stevioside 410 | MOL251830 | 12395712 | High | 2 |
| 411 | Farnesol 411 | MOL608579 | 8048477 | High | 5 |
| 412 | Oleic acid 412 | MOL882929 | 4158512 | High | 4 |
| 413 | Withaferin A 413 | MOL560470 | 4304703 | High | 4 |
| 414 | Colchicine 414 | MOL135709 | 2718301 | High | 5 |
| 415 | Docosahexaenoic acid (DHA) 415 | MOL691214 | 2854095 | High | 3 |
| 416 | ethyl vanillin 416 | MOL176799 | 14360657 | High | 5 |
| 417 | ethyl vanillin 417 | MOL929728 | 2170680 | High | 2 |
| 418 | Diosgenin 418 | MOL631474 | 14991667 | High | 5 |
| 419 | Diosgenin 419 | MOL953516 | 7441007 | High | 4 |
| 420 | Alanine derivative 420 | MOL795566 | 5052628 | High | 5 |
| 421 | Valine 421 | MOL462847 | 6886304 | High | 2 |
| 422 | Celastrol 422 | MOL302719 | 4373348 | High | 2 |
| 423 | caprylic acid 423 | MOL642383 | 2795153 | High | 5 |
| 424 | Stevioside 424 | MOL417379 | 2889079 | High | 5 |
| 425 | Betaine 425 | MOL727526 | 954098 | High | 3 |
| 426 | Silymarin 426 | MOL865721 | 7754459 | High | 5 |
| 427 | Salicin 427 | MOL394877 | 4122711 | High | 4 |
| 428 | Isoleucine 428 | MOL583596 | 4129947 | High | 2 |
| 429 | Valine derivative | MOL456787 | 5084509 | High | 5 |
| 430 | Baicalin | MOL140285 | 7121439 | High | 4 |
| 431 | Psoralen 431 | MOL553153 | 1271880 | High | 5 |
| 432 | Salicin 432 | MOL261684 | 4090287 | High | 3 |
| 433 | Gamma-Linolenic acid derivative | MOL529704 | 5500645 | High | 5 |
| 434 | vanillin derivative 434 | MOL417276 | 7057733 | High | 5 |
| 435 | Fisetin | MOL256570 | 12286602 | High | 3 |
| 436 | syringaldehyde 436 | MOL260876 | 1304613 | High | 2 |
| 437 | Linoleic acid 437 | MOL132593 | 4141908 | High | 5 |
| 438 | caprylic acid 438 | MOL344129 | 5606518 | High | 3 |
| 439 | Verbenone derivative 439 | MOL488065 | 5373342 | High | 5 |
| 440 | Carnosic acid 440 | MOL639361 | 1797132 | High | 2 |
| 441 | scopoletin 441 | MOL509672 | 14061026 | High | 4 |
| 442 | caproic acid 442 | MOL668749 | 960124 | High | 4 |
| 443 | aesculetin 443 | MOL810730 | 6620457 | High | 2 |
| 444 | Procyanidin B2 444 | MOL359996 | 10497465 | High | 2 |
| 445 | Taurine 445 | MOL397664 | 3091503 | High | 5 |
| 446 | Proline 446 | MOL678439 | 13494138 | High | 2 |
| 447 | Citronellol 447 | MOL102516 | 3281530 | High | 5 |
| 448 | ethyl vanillin 448 | MOL419060 | 13950123 | High | 2 |
| 449 | Saponin 449 | MOL328901 | 5090244 | High | 5 |
| 450 | Withaferin A 450 | MOL231422 | 12584398 | High | 3 |
| 451 | Matrine 451 | MOL654843 | 13990495 | High | 2 |
| 452 | Isoeugenol 452 | MOL864609 | 12741987 | High | 2 |
| 453 | Stevioside 453 | MOL387603 | 6781392 | High | 2 |
| 454 | Linalyl acetate 454 | MOL977600 | 6610440 | High | 5 |
| 455 | Tryptophan derivative | MOL453985 | 15020962 | High | 5 |
| 456 | Betaine 456 | MOL739332 | 1477258 | High | 2 |
| 457 | caproic acid 457 | MOL202117 | 4839761 | High | 3 |
| 458 | Parthenolide 458 | MOL837284 | 14853491 | High | 4 |
| 459 | Valine derivative 459 | MOL257088 | 15006406 | High | 5 |
| 460 | Andrographolide 460 | MOL325950 | 9134142 | High | 5 |
| 461 | Lupeol | MOL473146 | 14595357 | High | 5 |
| 462 | capric acid 462 | MOL300355 | 11897777 | High | 2 |
| 463 | Isoleucine 463 | MOL893463 | 4731809 | High | 5 |
| 464 | caproic acid 464 | MOL895814 | 3373158 | High | 2 |
| 465 | Eicosapentaenoic acid (EPA) 465 | MOL742818 | 1487694 | High | 4 |
| 466 | Linalyl acetate derivative 466 | MOL191965 | 10048063 | High | 3 |
| 467 | Carnosic acid 467 | MOL732460 | 1786521 | High | 2 |
| 468 | Creatine 468 | MOL891419 | 4806437 | High | 4 |
| 469 | Silymarin 469 | MOL123586 | 14465150 | High | 2 |
| 470 | scopoletin 470 | MOL776819 | 8763753 | High | 3 |
| 471 | Ellagic acid 471 | MOL760377 | 4441854 | High | 3 |
| 472 | Linoleic acid 472 | MOL191895 | 7050032 | High | 4 |
| 473 | Docosahexaenoic acid (DHA) derivative | MOL117475 | 6128448 | High | 5 |
| 474 | Lecithin 474 | MOL209387 | 13987615 | High | 5 |
| 475 | aesculetin 475 | MOL530355 | 13472590 | High | 2 |
| 476 | Scutellarin 476 | MOL822290 | 13041937 | High | 4 |
| 477 | scopoletin 477 | MOL246963 | 6768134 | High | 3 |
| 478 | Patchouli alcohol 478 | MOL973659 | 7458152 | High | 3 |
| 479 | Farnesol 479 | MOL616331 | 5526857 | High | 2 |
| 480 | Chrysin 480 | MOL692119 | 13284982 | High | 4 |
| 481 | Verbenone 481 | MOL827244 | 7541260 | High | 5 |
| 482 | Tyrosine 482 | MOL114522 | 14283923 | High | 4 |
| 483 | Icariin derivative 483 | MOL458860 | 7942895 | High | 4 |
| 484 | Carnosic acid 484 | MOL289916 | 1278600 | High | 2 |
| 485 | Linalyl acetate 485 | MOL144324 | 9992074 | High | 4 |
| 486 | Gamma-Linolenic acid 486 | MOL973216 | 11201139 | High | 4 |
| 487 | Saponin 487 | MOL266159 | 10463328 | High | 5 |
| 488 | Isoeugenol derivative 488 | MOL454079 | 6628485 | High | 3 |
| 489 | caproic acid 489 | MOL151205 | 3379688 | High | 3 |
| 490 | Chrysin derivative | MOL186902 | 2782055 | High | 2 |
| 491 | Choline 491 | MOL578338 | 14977526 | High | 4 |
| 492 | Chrysin 492 | MOL990692 | 229453 | High | 3 |
| 493 | Silymarin 493 | MOL965417 | 2613395 | High | 4 |
| 494 | scopoletin 494 | MOL674482 | 8938787 | High | 5 |
| 495 | Icariin derivative 495 | MOL893940 | 10525212 | High | 2 |
| 496 | Colchicine 496 | MOL774045 | 1038960 | High | 4 |
| 497 | Taurine derivative | MOL932688 | 2947000 | High | 5 |
| 498 | Valine 498 | MOL658197 | 13967444 | High | 3 |
| 499 | Isoeugenol 499 | MOL291064 | 13607041 | High | 2 |
| 500 | Lecithin 500 | MOL923191 | 9849067 | High | 2 |
| 501 | Farnesol 501 | MOL565175 | 2020134 | High | 4 |
| 502 | Fisetin derivative 502 | MOL394150 | 13016097 | High | 4 |
| 503 | Ergothioneine 503 | MOL437005 | 681792 | High | 5 |
| 504 | syringaldehyde 504 | MOL685405 | 15052766 | High | 5 |
| 505 | Geranyl acetate | MOL575083 | 4747099 | High | 3 |
| 506 | Farnesol 506 | MOL570682 | 4036938 | High | 5 |
| 507 | Arachidonic acid 507 | MOL782639 | 6101353 | High | 2 |
| 508 | Myricetin 508 | MOL618299 | 11857910 | High | 3 |
| 509 | ethyl vanillin 509 | MOL843046 | 10412624 | High | 2 |
| 510 | Arachidonic acid 510 | MOL928023 | 15025283 | High | 5 |
| 511 | Threonine 511 | MOL468423 | 11593251 | High | 2 |
| 512 | Stearic acid 512 | MOL581795 | 14794262 | High | 5 |
| 513 | Artemisinin 513 | MOL976085 | 7679357 | High | 5 |
| 514 | Isoleucine 514 | MOL193581 | 4373987 | High | 3 |
| 515 | Stearic acid derivative | MOL776165 | 10856982 | High | 3 |
| 516 | Alpha-Bisabolol | MOL485878 | 14403979 | High | 3 |
| 517 | Valine | MOL429682 | 520378 | High | 2 |
| 518 | Glutamic acid | MOL812864 | 12962299 | High | 4 |
| 519 | Scutellarin | MOL897404 | 14640146 | High | 5 |
| 520 | Glutathione | MOL986878 | 13438746 | High | 3 |
| 521 | Asparagine | MOL609065 | 6882578 | High | 2 |
| 522 | Scutellarin | MOL890956 | 1048463 | High | 3 |
| 523 | Hesperetin | MOL539637 | 7753775 | High | 5 |
| 524 | Gamma-Linolenic acid metabolite | MOL937141 | 2402557 | High | 5 |
| 525 | Carnosic acid | MOL943367 | 14218162 | High | 3 |
| 526 | Alpha-Linolenic acid | MOL730776 | 6982196 | High | 2 |
| 527 | Betaine | MOL914382 | 1755933 | High | 5 |
| 528 | Scutellarin | MOL336542 | 7305494 | High | 5 |
| 529 | Citronellol | MOL388236 | 10838777 | High | 4 |
| 530 | Alpha-Linolenic acid | MOL213019 | 4052476 | High | 2 |
| 531 | Zearalenone | MOL828091 | 1676787 | High | 3 |
| 532 | Withaferin A | MOL167647 | 7767656 | High | 3 |
| 533 | Threonine | MOL232550 | 7220419 | High | 3 |
| 534 | Echinacoside | MOL571131 | 4533169 | High | 2 |
| 535 | Lauric acid | MOL991136 | 2441353 | High | 5 |
| 536 | Triptolide | MOL374375 | 12530221 | High | 3 |
| 537 | Arachidonic acid | MOL167824 | 4734333 | High | 3 |
| 538 | Alpha-Bisabolol | MOL612550 | 7236688 | High | 5 |
| 539 | Choline | MOL805014 | 3088440 | High | 4 |
| 540 | Isoeugenol metabolite | MOL899405 | 3248731 | High | 3 |
| 541 | Artemisinin | MOL968347 | 9461202 | High | 4 |
| 542 | Linoleic acid | MOL635979 | 6872317 | High | 2 |
| 543 | Glutathione | MOL268508 | 9307951 | High | 5 |
| 544 | Lauric acid | MOL957290 | 4082620 | High | 3 |
| 545 | coumarin metabolite | MOL516685 | 2024155 | High | 5 |
| 546 | Valine | MOL527224 | 351762 | High | 5 |
| 547 | Triptolide | MOL952935 | 390671 | High | 5 |
| 548 | Echinacoside metabolite | MOL735334 | 13801742 | High | 4 |
| 549 | Hesperetin | MOL171880 | 14259803 | High | 2 |
| 550 | Stevioside | MOL922066 | 648288 | High | 3 |
| 551 | Scutellarin | MOL355647 | 10374619 | High | 4 |
| 552 | Hesperetin | MOL578685 | 10850247 | High | 3 |
| 553 | Leucine | MOL590086 | 258333 | High | 4 |
| 554 | Salicin | MOL358897 | 2367652 | High | 5 |
| 555 | Myristic acid | MOL969915 | 1787794 | High | 4 |
| 556 | Taxifolin metabolite | MOL245971 | 1543264 | High | 4 |
| 557 | caproic acid | MOL279598 | 3145976 | High | 2 |
| 558 | Alpha-Bisabolol | MOL453617 | 12446476 | High | 2 |
| 559 | Sinomenine | MOL538571 | 822994 | High | 3 |
| 560 | Fisetin | MOL584064 | 5338831 | High | 4 |
| 561 | Isoleucine | MOL455323 | 13227197 | High | 2 |
| 562 | Matrine | MOL250148 | 13089570 | High | 2 |
| 563 | Creatine | MOL409018 | 10598709 | High | 4 |
| 564 | Taurine | MOL174143 | 5581526 | High | 5 |
| 565 | caproic acid | MOL552723 | 10721387 | High | 3 |
| 566 | Inositol | MOL687001 | 11395421 | High | 5 |
| 567 | coumarin | MOL699403 | 483011 | High | 4 |
| 568 | vanillin metabolite | MOL739828 | 3933679 | High | 4 |
| 569 | Colchicine | MOL638428 | 7528947 | High | 5 |
| 570 | Salicin metabolite | MOL673346 | 14428030 | High | 2 |
| 571 | Taurine | MOL392498 | 3347353 | High | 2 |
| 572 | Zearalenone | MOL587011 | 14196540 | High | 3 |
| 573 | Alpha-Linolenic acid | MOL665443 | 2593671 | High | 2 |
| 574 | Tryptophan | MOL988283 | 4180084 | High | 4 |
| 575 | Citronellol | MOL821326 | 5079552 | High | 5 |
| 576 | Cedrol | MOL599974 | 13603499 | High | 2 |
| 577 | Glycine | MOL886463 | 5001280 | High | 2 |
| 578 | Geranyl acetate | MOL429414 | 7437285 | High | 3 |
| 579 | Andrographolide | MOL679040 | 5090101 | High | 3 |
| 580 | Withaferin A | MOL787404 | 2827636 | High | 5 |
| 581 | Psoralen | MOL122150 | 14781959 | High | 2 |
| 582 | ethyl vanillin | MOL686006 | 9169172 | High | 5 |
| 583 | Stearic acid | MOL777448 | 14506186 | High | 4 |
| 584 | Verbenone | MOL848638 | 9436689 | High | 2 |
| 585 | Proline | MOL117316 | 13609200 | High | 5 |
| 586 | Farnesol | MOL992595 | 9903923 | High | 5 |
| 587 | Colchicine | MOL631485 | 9673321 | High | 2 |
| 588 | Lupeol | MOL766476 | 8700506 | High | 3 |
| 589 | Yohimbine | MOL770686 | 633512 | High | 3 |
| 590 | Silymarin | MOL759369 | 5876297 | High | 3 |
| 591 | caproic acid metabolite | MOL936375 | 11081588 | High | 5 |
| 592 | Creatine | MOL547620 | 9009486 | High | 3 |
| 593 | Lauric acid | MOL528612 | 6420174 | High | 5 |
| 594 | Docosahexaenoic acid (DHA) | MOL171675 | 2730565 | High | 5 |
| 595 | Threonine | MOL565892 | 14518417 | High | 5 |
| 596 | Docosahexaenoic acid (DHA) | MOL966091 | 13752703 | High | 2 |
| 597 | Patchouli alcohol | MOL194212 | 13691115 | High | 3 |
| 598 | Baicalin | MOL524320 | 13539681 | High | 2 |
| 599 | Myristic acid | MOL650025 | 250679 | High | 4 |
| 600 | vanillin | MOL937078 | 6829067 | High | 5 |
| 601 | Taxifolin | MOL540154 | 14471954 | High | 2 |
| 602 | Alpha-Bisabolol | MOL439688 | 10308085 | High | 5 |
| 603 | coumarin | MOL786883 | 4433753 | High | 3 |
| 604 | Citronellol | MOL736148 | 5260280 | High | 4 |
| 605 | Alpha-Bisabolol | MOL805100 | 3089249 | High | 3 |
| 606 | Myricetin | MOL912344 | 11372336 | High | 4 |
| 607 | Xanthohumol | MOL186424 | 10517803 | High | 2 |
| 608 | Inositol | MOL367021 | 10725810 | High | 5 |
| 609 | Celastrol metabolite | MOL498602 | 9313298 | High | 3 |
| 610 | Osthole | MOL493805 | 10824383 | High | 3 |
| 611 | Icariin | MOL166416 | 11139396 | High | 5 |
| 612 | Procyanidin B2 metabolite | MOL641542 | 14922653 | High | 2 |
| 613 | Myricetin | MOL415662 | 7424893 | High | 5 |
| 614 | Cedrol | MOL761551 | 7897154 | High | 3 |
| 615 | Lupeol | MOL863760 | 1486163 | High | 2 |
| 616 | Glycine | MOL547371 | 4138824 | High | 5 |
| 617 | Lecithin metabolite | MOL848255 | 4994234 | High | 3 |
| 618 | caproic acid | MOL421203 | 3559565 | High | 3 |
| 619 | Eicosapentaenoic acid (EPA) | MOL934453 | 1713020 | High | 3 |
| 620 | Taxifolin | MOL438904 | 5446954 | High | 5 |
| 621 | Taxifolin metabolite | MOL978970 | 1254755 | High | 4 |
| 622 | Choline | MOL280278 | 11660330 | High | 4 |
| 623 | Serine metabolite | MOL789621 | 9982253 | High | 5 |
| 624 | Matrine | MOL326832 | 10597033 | High | 2 |
| 625 | Andrographolide | MOL379444 | 4964170 | High | 5 |
| 626 | Yohimbine | MOL813712 | 2426036 | High | 3 |
| 627 | Artemisinin | MOL133549 | 12571258 | High | 3 |
| 628 | caprylic acid | MOL619703 | 12280108 | High | 4 |
| 629 | Alpha-Linolenic acid | MOL297390 | 13276864 | High | 3 |
| 630 | Stearic acid | MOL909590 | 15075396 | High | 5 |
| 631 | Scutellarin | MOL270931 | 12701760 | High | 4 |
| 632 | Taxifolin | MOL429805 | 7914037 | High | 3 |
| 633 | caprylic acid | MOL589190 | 7634113 | High | 3 |
| 634 | Glutamine | MOL538132 | 4526561 | High | 5 |
| 635 | Oleic acid | MOL873250 | 12283161 | High | 5 |
| 636 | Arginine | MOL655273 | 14844163 | High | 2 |
| 637 | Chrysin | MOL201411 | 10105144 | High | 3 |
| 638 | Geranyl acetate | MOL751672 | 4616869 | High | 5 |
| 639 | Taurine | MOL493802 | 12232117 | High | 5 |
| 640 | Serine | MOL394859 | 2681737 | High | 5 |
| 641 | capric acid | MOL398196 | 14394700 | High | 3 |
| 642 | Echinacoside metabolite | MOL953024 | 5169688 | High | 2 |
| 643 | Patchouli alcohol | MOL365141 | 4658136 | High | 2 |
| 644 | butyric acid metabolite | MOL511885 | 9310079 | High | 4 |
| 645 | Serine | MOL235270 | 9871092 | High | 4 |
| 646 | Cedrol | MOL552595 | 1947607 | High | 5 |
| 647 | vanillin | MOL743281 | 10919665 | High | 3 |
| 648 | Triptolide metabolite | MOL328685 | 9668639 | High | 5 |
| 649 | Myristic acid | MOL505150 | 5279985 | High | 5 |
| 650 | Linalyl acetate | MOL710351 | 15071132 | High | 3 |
| 651 | Chrysin metabolite | MOL850327 | 6620967 | High | 4 |
| 652 | Eicosapentaenoic acid (EPA) metabolite | MOL489549 | 1460818 | High | 5 |
| 653 | Patchouli alcohol | MOL514312 | 8424262 | High | 4 |
| 654 | Andrographolide | MOL527681 | 943415 | High | 2 |
| 655 | Zearalenone | MOL882351 | 2675032 | High | 4 |
| 656 | Linoleic acid | MOL448143 | 3633541 | High | 3 |
| 657 | Osthole metabolite | MOL316315 | 11198278 | High | 5 |
| 658 | Carnosic acid | MOL419589 | 6753741 | High | 5 |
| 659 | Inositol | MOL245300 | 11581926 | High | 5 |
| 660 | Andrographolide | MOL799192 | 11214081 | High | 3 |
| 661 | Geranyl acetate | MOL635226 | 3291345 | High | 5 |
| 662 | Procyanidin B2 | MOL145774 | 4154442 | High | 5 |
| 663 | Yohimbine | MOL650481 | 9435574 | High | 5 |
| 664 | Procyanidin B2 | MOL479180 | 9901075 | High | 5 |
| 665 | Farnesol | MOL832895 | 2588879 | High | 5 |
| 666 | Cedrol metabolite | MOL648352 | 4911273 | High | 5 |
| 667 | scopoletin | MOL387608 | 8274817 | High | 3 |
| 668 | Myricetin | MOL190504 | 13222885 | High | 5 |
| 669 | Stearic acid metabolite | MOL955132 | 14923291 | High | 2 |
| 670 | Lupeol | MOL583467 | 12497923 | High | 3 |
| 671 | Tryptophan | MOL542710 | 9726639 | High | 4 |
| 672 | Terpinen-4-ol metabolite | MOL811612 | 4335503 | High | 5 |
| 673 | Terpinen-4-ol metabolite | MOL289803 | 8787507 | High | 2 |
| 674 | scopoletin | MOL310274 | 10147001 | High | 3 |
| 675 | Arginine metabolite | MOL970226 | 13034461 | High | 5 |
| 676 | Lecithin | MOL564798 | 405090 | High | 5 |
| 677 | Silymarin | MOL867190 | 1494715 | High | 2 |
| 678 | Myristic acid | MOL964319 | 9607507 | High | 3 |
| 679 | Triptolide | MOL645907 | 7121058 | High | 4 |
| 680 | Hesperetin | MOL209879 | 13559173 | High | 5 |
| 681 | Betaine | MOL474675 | 11506764 | High | 2 |
| 682 | Eicosapentaenoic acid (EPA) | MOL328138 | 14087689 | High | 2 |
| 683 | Threonine | MOL979368 | 10194591 | High | 5 |
| 684 | Triptolide metabolite | MOL949980 | 5074355 | High | 5 |
| 685 | Farnesol | MOL783207 | 13948505 | High | 5 |
| 686 | Vincristine | MOL882862 | 11571163 | High | 4 |
| 687 | Farnesol metabolite | MOL545999 | 10259755 | High | 4 |
| 688 | Ergothioneine metabolite | MOL374413 | 1986765 | High | 3 |
| 689 | Linoleic acid | MOL254131 | 6047297 | High | 3 |
| 690 | Andrographolide | MOL969750 | 7638975 | High | 5 |
| 691 | Scutellarin | MOL370535 | 1042384 | High | 5 |
| 692 | Vincristine | MOL508171 | 11006162 | High | 5 |
| 693 | Valine | MOL423935 | 8454195 | High | 4 |
| 694 | butyric acid metabolite | MOL355803 | 8953552 | High | 3 |
| 695 | Chrysin | MOL792744 | 12549830 | High | 4 |
| 696 | Myricetin | MOL892632 | 5314653 | High | 4 |
| 697 | Betaine | MOL337097 | 14185976 | High | 4 |
| 698 | vanillin | MOL631388 | 4471314 | High | 3 |
| 699 | Lupeol metabolite | MOL992500 | 350037 | High | 4 |
| 700 | Stearic acid | MOL878611 | 9867494 | High | 3 |
| 701 | Alanine | MOL306530 | 3073308 | High | 3 |
| 702 | Leucine | MOL804442 | 12878301 | High | 4 |
| 703 | Lecithin | MOL395069 | 5447097 | High | 3 |
| 704 | Baicalin | MOL775272 | 12887181 | High | 3 |
| 705 | Alpha-Linolenic acid | MOL850354 | 4817001 | High | 2 |
| 706 | Saponin | MOL318099 | 3850055 | High | 2 |
| 707 | Cedrol metabolite | MOL863786 | 14103532 | High | 2 |
| 708 | Scutellarin | MOL924196 | 6150194 | High | 5 |
| 709 | Andrographolide | MOL740908 | 8397587 | High | 2 |
| 710 | Proline | MOL417176 | 8671000 | High | 2 |
| 711 | Taurine | MOL131677 | 2424287 | High | 5 |
| 712 | Isoleucine | MOL404593 | 144077 | High | 3 |
| 713 | Isoleucine | MOL681209 | 5894552 | High | 4 |
| 714 | Asparagine metabolite | MOL569353 | 7067299 | High | 5 |
| 715 | Alpha-Linolenic acid | MOL731857 | 13733406 | High | 3 |
| 716 | Plumbagin metabolite | MOL589863 | 9817315 | High | 3 |
| 717 | Oleic acid | MOL172851 | 12538059 | High | 4 |
| 718 | Farnesol metabolite | MOL800644 | 2166590 | High | 3 |
| 719 | Phloretin | MOL129745 | 3861251 | High | 5 |
| 720 | Parthenolide | MOL250289 | 11881885 | High | 5 |
| 721 | Lecithin metabolite | MOL750844 | 4326766 | High | 3 |
| 722 | Verbenone | MOL259593 | 7609100 | High | 2 |
| 723 | Taurine metabolite | MOL168280 | 4412215 | High | 4 |
| 724 | Baicalin | MOL127542 | 5098592 | High | 3 |
| 725 | Echinacoside | MOL519314 | 3615110 | High | 3 |
| 726 | Withaferin A | MOL702201 | 10442537 | High | 2 |
| 727 | Nerolidol | MOL894353 | 14709302 | High | 4 |
| 728 | Hesperetin | MOL593496 | 6531786 | High | 5 |
| 729 | Xanthohumol | MOL799434 | 5204852 | High | 2 |
| 730 | Phloretin | MOL150833 | 14148167 | High | 2 |
| 731 | Eicosapentaenoic acid (EPA) | MOL907060 | 11658143 | High | 4 |
| 732 | Lupeol | MOL893722 | 13735516 | High | 5 |
| 733 | Borneol metabolite | MOL163425 | 9360392 | High | 4 |
| 734 | Terpinen-4-ol | MOL808383 | 3736132 | High | 5 |
| 735 | Icariin | MOL806848 | 3693170 | High | 3 |
| 736 | Procyanidin B2 metabolite | MOL412860 | 10692909 | High | 5 |
| 737 | Lauric acid | MOL729373 | 1499329 | High | 5 |
| 738 | Arachidonic acid | MOL128967 | 4264819 | High | 3 |
| 739 | Galangin metabolite | MOL961112 | 4128646 | High | 3 |
| 740 | Arachidonic acid | MOL251356 | 6918527 | High | 5 |
| 741 | scopoletin | MOL919561 | 13464069 | High | 3 |
| 742 | Citronellol | MOL917567 | 2069009 | High | 5 |
| 743 | Alpha-Linolenic acid | MOL441618 | 2150774 | High | 3 |
| 744 | Zearalenone | MOL733637 | 6733372 | High | 2 |
| 745 | Proline metabolite | MOL600396 | 639257 | High | 2 |
| 746 | Citronellol | MOL845792 | 225829 | High | 4 |
| 747 | Saponin | MOL847867 | 12456934 | High | 5 |
| 748 | Psoralen | MOL962329 | 1502399 | High | 3 |
| 749 | Glutamine | MOL783379 | 4597572 | High | 2 |
| 750 | Citronellol | MOL467549 | 9148920 | High | 4 |
| 751 | Lecithin | MOL774152 | 12338604 | High | 2 |
| 752 | Xanthohumol | MOL637825 | 9261711 | High | 5 |
| 753 | syringaldehyde | MOL591749 | 4221285 | High | 4 |
| 754 | Linoleic acid | MOL417783 | 13665982 | High | 2 |
| 755 | Creatine | MOL963666 | 585013 | High | 2 |
| 756 | Phloretin | MOL765721 | 14410933 | High | 3 |
| 757 | Serine | MOL707367 | 1653376 | High | 2 |
| 758 | Docosahexaenoic acid (DHA) | MOL875512 | 6988920 | High | 5 |
| 759 | Verbenone | MOL114457 | 10447475 | High | 2 |
| 760 | Lecithin | MOL414013 | 15028779 | High | 2 |
| 761 | Parthenolide | MOL328065 | 1103027 | High | 5 |
| 762 | Farnesol | MOL807941 | 13700547 | High | 5 |
| 763 | Celastrol | MOL653771 | 9280870 | High | 3 |
| 764 | caproic acid | MOL994130 | 10921081 | High | 2 |
| 765 | ethyl vanillin | MOL973435 | 11605050 | High | 5 |
| 766 | Vincristine | MOL293641 | 9398783 | High | 3 |
| 767 | Cedrol | MOL159604 | 1247605 | High | 4 |
| 768 | Glutamine metabolite | MOL411171 | 4978160 | High | 4 |
| 769 | coumarin | MOL557763 | 11323979 | High | 2 |
| 770 | caprylic acid metabolite | MOL262812 | 14924283 | High | 2 |
| 771 | Betaine | MOL265001 | 5188335 | High | 2 |
| 772 | Carnosic acid | MOL758551 | 12628254 | High | 4 |
| 773 | Glycine | MOL856282 | 12808939 | High | 5 |
| 774 | Linalyl acetate | MOL869430 | 14178956 | High | 4 |
| 775 | Icariin | MOL474140 | 14667114 | High | 3 |
| 776 | Alpha-Bisabolol | MOL430410 | 11111227 | High | 3 |
| 777 | Stevioside | MOL733833 | 970003 | High | 4 |
| 778 | capric acid | MOL127327 | 10331506 | High | 3 |
| 779 | Eicosapentaenoic acid (EPA) metabolite | MOL600916 | 5312558 | High | 2 |
| 780 | Stevioside metabolite | MOL919615 | 10016474 | High | 3 |
| 781 | Arginine | MOL904539 | 1274097 | High | 3 |
| 782 | Alanine | MOL928112 | 8489939 | High | 2 |
| 783 | Xanthohumol metabolite | MOL359616 | 3669246 | High | 5 |
| 784 | Farnesol | MOL828340 | 1110287 | High | 2 |
| 785 | Phloretin | MOL191702 | 12429561 | High | 2 |
| 786 | Glutathione | MOL640613 | 4995794 | High | 2 |
| 787 | Betaine | MOL681628 | 4535505 | High | 3 |
| 788 | Vincristine | MOL598469 | 5999995 | High | 2 |
| 789 | Xanthohumol | MOL783063 | 13210183 | High | 5 |
| 790 | Leucine | MOL632024 | 1690959 | High | 2 |
| 791 | Creatine | MOL677181 | 7552026 | High | 3 |
| 792 | Phloretin metabolite | MOL355694 | 7258306 | High | 4 |
| 793 | syringaldehyde metabolite | MOL311060 | 1419836 | High | 3 |
| 794 | Asparagine metabolite | MOL171583 | 7619110 | High | 2 |
| 795 | Valine | MOL279304 | 9996826 | High | 2 |
| 796 | Alpha-Linolenic acid | MOL978712 | 7069056 | High | 3 |
| 797 | Salicin | MOL486475 | 8427330 | High | 2 |
| 798 | caprylic acid | MOL503722 | 501269 | High | 4 |
| 799 | Diosgenin | MOL981160 | 9687785 | High | 4 |
| 800 | Glycine metabolite | MOL361036 | 4057704 | High | 4 |
| 801 | Carnosic acid | MOL836536 | 6537513 | High | 5 |
| 802 | Baicalin metabolite | MOL193244 | 11319972 | High | 3 |
| 803 | Psoralen metabolite | MOL641636 | 13263031 | High | 2 |
| 804 | Zearalenone | MOL908072 | 12441627 | High | 2 |
| 805 | Artemisinin | MOL722231 | 2947381 | High | 2 |
| 806 | Cedrol | MOL901698 | 1561974 | High | 4 |
| 807 | vanillin | MOL698099 | 2605609 | High | 2 |
| 808 | Geranyl acetate metabolite | MOL501450 | 8462918 | High | 5 |
| 809 | Withaferin A | MOL784159 | 10616094 | High | 3 |
| 810 | Icariin | MOL180026 | 12263041 | High | 5 |
| 811 | Hesperetin | MOL767210 | 193777 | High | 2 |
| 812 | Cedrol | MOL768309 | 5034207 | High | 3 |
| 813 | Oleic acid | MOL406552 | 14654742 | High | 2 |
| 814 | Linoleic acid metabolite | MOL599411 | 1447126 | High | 3 |
| 815 | Lauric acid | MOL354370 | 9853123 | High | 4 |
| 816 | Stearic acid | MOL646667 | 9326109 | High | 4 |
| 817 | Tryptophan metabolite | MOL400464 | 13640220 | High | 2 |
| 818 | Alanine | MOL699385 | 12896803 | High | 3 |
| 819 | caprylic acid metabolite | MOL760204 | 7405050 | High | 4 |
| 820 | Lauric acid metabolite | MOL984513 | 12391846 | High | 5 |
| 821 | aesculetin | MOL729056 | 4516365 | High | 3 |
| 822 | Terpinen-4-ol metabolite | MOL800143 | 1682834 | High | 3 |
| 823 | Parthenolide | MOL303336 | 14397489 | High | 3 |
| 824 | Alpha-Linolenic acid | MOL557189 | 2555634 | High | 2 |
| 825 | Alpha-Linolenic acid | MOL564990 | 3002196 | High | 4 |
| 826 | coumarin | MOL702472 | 3336357 | High | 2 |
| 827 | Stevioside | MOL607517 | 11058400 | High | 5 |
| 828 | capric acid | MOL199906 | 9009819 | High | 3 |
| 829 | Xanthohumol | MOL934327 | 4954762 | High | 5 |
| 830 | Yohimbine | MOL925345 | 3202991 | High | 4 |
| 831 | Procyanidin B2 | MOL244991 | 3212355 | High | 3 |
| 832 | Taurine metabolite | MOL136471 | 3263633 | High | 5 |
| 833 | Alanine | MOL362929 | 11232334 | High | 2 |
| 834 | Procyanidin B2 | MOL974003 | 2519493 | High | 2 |
| 835 | Alanine metabolite | MOL977190 | 2893964 | High | 2 |
| 836 | Taxifolin | MOL303728 | 6752459 | High | 5 |
| 837 | Verbenone metabolite | MOL700863 | 3962538 | High | 2 |
| 838 | Serine | MOL861026 | 8343730 | High | 5 |
| 839 | Gamma-Linolenic acid | MOL687867 | 6679219 | High | 2 |
| 840 | Vincristine | MOL986744 | 8788068 | High | 5 |
| 841 | Xanthohumol | MOL451671 | 6994418 | High | 5 |
| 842 | Lecithin | MOL640278 | 13815141 | High | 2 |
| 843 | Asparagine | MOL357567 | 6805501 | High | 5 |
| 844 | coumarin metabolite | MOL197025 | 6261500 | High | 3 |
| 845 | Terpinen-4-ol metabolite | MOL191528 | 7297167 | High | 5 |
| 846 | Choline metabolite | MOL499153 | 483564 | High | 3 |
| 847 | Tryptophan | MOL797848 | 10636539 | High | 3 |
| 848 | Alpha-Bisabolol | MOL759018 | 3878711 | High | 5 |
| 849 | Betaine | MOL341963 | 10297060 | High | 2 |
| 850 | Alpha-Bisabolol | MOL293657 | 5905242 | High | 2 |
| 851 | Matrine metabolite | MOL497117 | 4983250 | High | 4 |
| 852 | Tryptophan | MOL181114 | 8906761 | High | 5 |
| 853 | Asparagine | MOL322729 | 5510354 | High | 5 |
| 854 | Tryptophan | MOL769146 | 12807953 | High | 4 |
| 855 | Taxifolin | MOL484167 | 10646889 | High | 5 |
| 856 | Choline metabolite | MOL912839 | 4998134 | High | 5 |
| 857 | Taxifolin | MOL914904 | 5290381 | High | 3 |
| 858 | Alpha-Linolenic acid | MOL987192 | 8372107 | High | 4 |
| 859 | syringaldehyde | MOL408078 | 5481382 | High | 2 |
| 860 | Stearic acid metabolite | MOL973955 | 14872827 | High | 3 |
| 861 | Zearalenone | MOL759475 | 5384125 | High | 5 |
| 862 | Taxifolin | MOL554988 | 2111141 | High | 5 |
| 863 | Lupeol | MOL471278 | 2046062 | High | 2 |
| 864 | Scutellarin | MOL844868 | 7996415 | High | 4 |
| 865 | Diosgenin | MOL805327 | 13127418 | High | 3 |
| 866 | Citronellol | MOL507530 | 443791 | High | 3 |
| 867 | Betaine | MOL762889 | 8590975 | High | 4 |
| 868 | Chrysin | MOL885245 | 11097088 | High | 2 |
| 869 | Triptolide metabolite | MOL359345 | 783478 | High | 3 |
| 870 | Inositol | MOL465012 | 11630786 | High | 5 |
| 871 | Echinacoside | MOL954062 | 7441831 | High | 5 |
| 872 | Parthenolide metabolite | MOL386055 | 8296612 | High | 4 |
| 873 | Salicin | MOL188664 | 8929051 | High | 5 |
| 874 | Ergothioneine metabolite | MOL429024 | 11549628 | High | 4 |
| 875 | Yohimbine | MOL755198 | 5622611 | High | 2 |
| 876 | aesculetin metabolite | MOL111933 | 3989801 | High | 3 |
| 877 | Echinacoside metabolite | MOL573344 | 11143392 | High | 2 |
| 878 | Echinacoside | MOL769495 | 1350608 | High | 2 |
| 879 | Verbenone | MOL501117 | 9981213 | High | 4 |
| 880 | Borneol | MOL802406 | 3851903 | High | 3 |
| 881 | Xanthohumol | MOL194719 | 13089227 | High | 2 |
| 882 | Carnosic acid | MOL796534 | 10159480 | High | 2 |
| 883 | Citronellol | MOL820017 | 258548 | High | 3 |
| 884 | Myricetin | MOL962339 | 342402 | High | 2 |
| 885 | Eicosapentaenoic acid (EPA) | MOL400523 | 196649 | High | 4 |
| 886 | Plumbagin | MOL584795 | 8139062 | High | 3 |
| 887 | Salicin metabolite | MOL606440 | 12296591 | High | 4 |
| 888 | Threonine | MOL457633 | 7214575 | High | 3 |
| 889 | Taxifolin | MOL789845 | 10996830 | High | 4 |
| 890 | Saponin | MOL793798 | 936240 | High | 3 |
| 891 | Choline | MOL139959 | 14387554 | High | 5 |
| 892 | Xanthohumol | MOL192151 | 11817139 | High | 5 |
| 893 | aesculetin | MOL863191 | 5560848 | High | 2 |
| 894 | Oleic acid metabolite | MOL584410 | 12179424 | High | 2 |
| 895 | Alpha-Bisabolol | MOL754752 | 7868782 | High | 4 |
| 896 | Geranyl acetate | MOL644483 | 559446 | High | 4 |
| 897 | Myristic acid metabolite | MOL934019 | 10664493 | High | 4 |
| 898 | Echinacoside | MOL436866 | 12406133 | High | 2 |
| 899 | Colchicine | MOL111346 | 13339633 | High | 4 |
| 900 | Xanthohumol | MOL705777 | 4893570 | High | 2 |
| 901 | Myricetin | MOL418469 | 5230081 | High | 2 |
| 902 | Betaine | MOL283237 | 13169523 | High | 2 |
| 903 | Silymarin | MOL688866 | 11241546 | High | 5 |
| 904 | Salicin | MOL139636 | 8447120 | High | 2 |
| 905 | Xanthohumol | MOL123290 | 9031134 | High | 4 |
| 906 | Myricetin | MOL300301 | 14778083 | High | 5 |
| 907 | Threonine | MOL919209 | 7316534 | High | 2 |
| 908 | Isoeugenol | MOL467795 | 2765064 | High | 4 |
| 909 | Arachidonic acid | MOL315388 | 871791 | High | 3 |
| 910 | butyric acid metabolite | MOL437686 | 2233650 | High | 2 |
| 911 | ethyl vanillin | MOL417614 | 3396885 | High | 5 |
| 912 | Isoleucine | MOL591080 | 5512040 | High | 4 |
| 913 | Triptolide | MOL621098 | 11384015 | High | 3 |
| 914 | Salicin | MOL496609 | 11974754 | High | 4 |
| 915 | Withaferin A | MOL856665 | 10698612 | High | 5 |
| 916 | Andrographolide | MOL549595 | 4734827 | High | 4 |
| 917 | Chrysin | MOL181166 | 12010206 | High | 3 |
| 918 | Linalyl acetate | MOL859888 | 6622449 | High | 2 |
| 919 | Phloretin | MOL666340 | 10339169 | High | 3 |
| 920 | Plumbagin | MOL233438 | 4262650 | High | 3 |
| 921 | Saponin metabolite | MOL836865 | 2756436 | High | 2 |
| 922 | Tryptophan | MOL731054 | 2185603 | High | 2 |
| 923 | Baicalin | MOL923329 | 13478906 | High | 3 |
| 924 | Inositol | MOL569480 | 8509609 | High | 4 |
| 925 | Psoralen metabolite | MOL931675 | 14087853 | High | 3 |
| 926 | Withaferin A metabolite | MOL464438 | 2122195 | High | 3 |
| 927 | Artemisinin | MOL176438 | 10166515 | High | 2 |
| 928 | aesculetin | MOL845013 | 7530230 | High | 2 |
| 929 | Linoleic acid | MOL808116 | 6960985 | High | 4 |
| 930 | Saponin metabolite | MOL332489 | 10531514 | High | 4 |
| 931 | Linoleic acid | MOL653229 | 15070487 | High | 5 |
| 932 | Psoralen | MOL853733 | 10043390 | High | 4 |
| 933 | Taxifolin metabolite | MOL334135 | 3774552 | High | 2 |
| 934 | Patchouli alcohol | MOL745279 | 11440342 | High | 2 |
| 935 | Chrysin metabolite | MOL913070 | 5161407 | High | 4 |
| 936 | Psoralen | MOL241948 | 14554731 | High | 2 |
| 937 | Echinacoside metabolite | MOL291389 | 13978817 | High | 5 |
| 938 | caproic acid | MOL321416 | 9959735 | High | 2 |
| 939 | Xanthohumol | MOL285828 | 12158069 | High | 4 |
| 940 | Artemisinin | MOL711750 | 9398632 | High | 4 |
| 941 | Linoleic acid | MOL981564 | 11221220 | High | 2 |
| 942 | Yohimbine | MOL768172 | 7798127 | High | 5 |
| 943 | Sinomenine | MOL677180 | 1614192 | High | 5 |
| 944 | Citronellol | MOL331490 | 9812223 | High | 3 |
| 945 | Colchicine | MOL757001 | 14106356 | High | 5 |
| 946 | Carnosic acid metabolite | MOL100437 | 12662447 | High | 5 |
| 947 | Scutellarin | MOL671831 | 13126780 | High | 4 |
| 948 | Vincristine | MOL264822 | 8706488 | High | 4 |
| 949 | Patchouli alcohol metabolite | MOL188964 | 255192 | High | 5 |
| 950 | Terpinen-4-ol metabolite | MOL829954 | 3024348 | High | 5 |
| 951 | Yohimbine metabolite | MOL443062 | 11390745 | High | 4 |
| 952 | Linalyl acetate metabolite | MOL616291 | 6553065 | High | 2 |
| 953 | Stevioside | MOL420965 | 4868118 | High | 5 |
| 954 | Chrysin | MOL953694 | 1661162 | High | 5 |
| 955 | ethyl vanillin metabolite | MOL892061 | 11800530 | High | 3 |
| 956 | Osthole | MOL500372 | 2774434 | High | 3 |
| 957 | Tryptophan | MOL773363 | 13394282 | High | 4 |
| 958 | Ergothioneine | MOL996645 | 11835546 | High | 4 |
| 959 | Echinacoside | MOL982070 | 9328179 | High | 3 |
| 960 | Parthenolide | MOL765406 | 14965962 | High | 3 |
| 961 | caprylic acid | MOL754113 | 8586587 | High | 5 |
| 962 | Myristic acid | MOL316811 | 6924650 | High | 3 |
| 963 | Alpha-Linolenic acid | MOL323665 | 5653728 | High | 5 |
| 964 | coumarin | MOL327160 | 5280176 | High | 2 |
| 965 | Sinomenine | MOL319440 | 11858276 | High | 5 |
| 966 | Artemisinin metabolite | MOL664858 | 11972066 | High | 4 |
| 967 | Geranyl acetate | MOL626324 | 1673323 | High | 2 |
| 968 | Psoralen | MOL384651 | 619742 | High | 5 |
| 969 | Alpha-Linolenic acid | MOL879724 | 12278295 | High | 2 |
| 970 | Serine metabolite | MOL251845 | 894809 | High | 3 |
| 971 | capric acid | MOL276543 | 13035032 | High | 5 |
| 972 | Ellagic acid | MOL785940 | 7652460 | High | 4 |
| 973 | Phloretin | MOL925535 | 5542461 | High | 3 |
| 974 | Celastrol | MOL128123 | 5682310 | High | 5 |
| 975 | Linoleic acid | MOL512490 | 10010619 | High | 3 |
| 976 | Lupeol | MOL162304 | 13789950 | High | 4 |
| 977 | Parthenolide metabolite | MOL690958 | 5890689 | High | 2 |
| 978 | vanillin | MOL236656 | 5528610 | High | 4 |
| 979 | Salicin | MOL167551 | 12561991 | High | 2 |
| 980 | Geranyl acetate | MOL532741 | 12231967 | High | 3 |
| 981 | Proline | MOL378030 | 7729550 | High | 2 |
| 982 | Terpinen-4-ol | MOL559720 | 14539938 | High | 3 |
| 983 | Alanine metabolite | MOL804766 | 6867340 | High | 3 |
| 984 | Glutathione | MOL984967 | 13426685 | High | 2 |
| 985 | Alpha-Bisabolol | MOL918452 | 11739220 | High | 3 |
| 986 | Gamma-Linolenic acid | MOL454291 | 14162147 | High | 3 |
| 987 | Farnesol | MOL285136 | 2944143 | High | 3 |
| 988 | Colchicine | MOL281190 | 9483862 | High | 2 |
| 989 | Lupeol | MOL405520 | 1458949 | High | 5 |
| 990 | Ergothioneine metabolite | MOL777375 | 5770029 | High | 2 |
| 991 | Citronellol metabolite | MOL999074 | 14827585 | High | 2 |
| 992 | Withaferin A | MOL691622 | 10113439 | High | 4 |
| 993 | Psoralen | MOL514506 | 4112902 | High | 4 |
| 994 | Nerolidol | MOL288839 | 1686267 | High | 3 |
| 995 | Citronellol | MOL998450 | 12286661 | High | 4 |
| 996 | butyric acid metabolite | MOL558265 | 11585250 | High | 5 |
| 997 | capric acid | MOL921605 | 11426775 | High | 3 |
| 998 | Cedrol | MOL856374 | 3137117 | High | 2 |
| 999 | Gamma-Linolenic acid | MOL547126 | 981451 | High | 4 |
| 1000 | Isoleucine | MOL304139 | 11231512 | High | 5 |
| 1001 | Diosgenin | MOL991126 | 6316008 | High | 4 |
| 1002 | Parthenolide | MOL248475 | 5095565 | High | 4 |
| 1003 | Triptolide | MOL583129 | 773929 | High | 2 |
| 1004 | Psoralen | MOL981813 | 1195840 | High | 5 |
| 1005 | Silymarin metabolite | MOL209784 | 1058322 | High | 2 |
| 1006 | Glutamic acid | MOL900091 | 880330 | High | 4 |
| 1007 | caproic acid | MOL644620 | 4532873 | High | 3 |
| 1008 | Proline | MOL324194 | 8661291 | High | 4 |
| 1009 | Creatine | MOL105404 | 2041766 | High | 3 |
| 1010 | syringaldehyde | MOL229232 | 3944170 | High | 5 |
| 1011 | Salicin | MOL245793 | 7996655 | High | 4 |
| 1012 | caproic acid | MOL504015 | 5800589 | High | 5 |
| 1013 | Alanine metabolite | MOL198703 | 12939166 | High | 3 |
| 1014 | Tryptophan | MOL493443 | 1178250 | High | 5 |
| 1015 | Colchicine | MOL535226 | 4721376 | High | 5 |
| 1016 | Andrographolide | MOL662921 | 1969627 | High | 3 |
| 1017 | Patchouli alcohol | MOL230833 | 5909483 | High | 5 |
| 1018 | Glutathione metabolite | MOL210538 | 8063280 | High | 5 |
| 1019 | Isoeugenol | MOL781604 | 4292122 | High | 2 |
| 1020 | Alpha-Linolenic acid | MOL977940 | 13757018 | High | 5 |
| 1021 | Tyrosine | MOL606216 | 8878093 | High | 3 |
| 1022 | Betaine | MOL191427 | 6683115 | High | 5 |
| 1023 | Echinacoside | MOL335538 | 2111524 | High | 5 |
| 1024 | Lupeol | MOL373857 | 12535902 | High | 5 |
| 1025 | vanillin | MOL255402 | 7854688 | High | 5 |
| 1026 | Tyrosine | MOL420192 | 4353453 | High | 3 |
| 1027 | Vincristine | MOL790650 | 9901043 | High | 5 |
| 1028 | coumarin | MOL716342 | 10848065 | High | 3 |
| 1029 | Triptolide | MOL522521 | 6471307 | High | 5 |
| 1030 | Arachidonic acid | MOL204666 | 9588443 | High | 3 |
| 1031 | Stevioside | MOL131546 | 10872098 | High | 2 |
| 1032 | Colchicine | MOL870295 | 4353976 | High | 4 |
| 1033 | Verbenone | MOL384917 | 133715 | High | 2 |
| 1034 | ZINC87593164 | MOL548124 | 5824818 | High | 3 |
| 1035 | Carnosic acid | MOL741187 | 9947553 | High | 2 |
| 1036 | Lupeol | MOL658730 | 8504508 | High | 2 |
| 1037 | Choline | MOL438491 | 216530 | High | 5 |
| 1038 | Scutellarin | MOL617733 | 12386397 | High | 5 |
| 1039 | ethyl vanillin | MOL621315 | 5687764 | High | 4 |
| 1040 | ethyl vanillin | MOL615998 | 9896000 | High | 3 |
| 1041 | Linoleic acid isomer | MOL676975 | 1590999 | High | 3 |
| 1042 | Saponin | MOL546610 | 11652820 | High | 5 |
| 1043 | Saponin | MOL252170 | 7340416 | High | 2 |
| 1044 | Stearic acid | MOL334123 | 9239984 | High | 3 |
| 1045 | Geranyl acetate | MOL685674 | 1037337 | High | 5 |
| 1046 | Lauric acid | MOL388261 | 3469959 | High | 3 |
| 1047 | Proline | MOL213094 | 5635353 | High | 5 |
| 1048 | Vincristine | MOL277798 | 11894593 | High | 2 |
| 1049 | Alanine | MOL572605 | 1739894 | High | 2 |
| 1050 | Lauric acid | MOL532530 | 8935094 | High | 5 |
| 1051 | Terpinen-4-ol | MOL586236 | 1210538 | High | 2 |
| 1052 | Myricetin | MOL823345 | 1095750 | High | 4 |
| 1053 | coumarin isomer | MOL104857 | 11099605 | High | 4 |
| 1054 | Artemisinin | MOL425626 | 14754098 | High | 4 |
| 1055 | Galangin | MOL472245 | 6051482 | High | 4 |
| 1056 | vanillin | MOL311830 | 12797817 | High | 3 |
| 1057 | Asparagine | MOL286406 | 13925274 | High | 4 |
| 1058 | Psoralen | MOL475832 | 3209768 | High | 4 |
| 1059 | Proline | MOL809622 | 12964459 | High | 2 |
| 1060 | Docosahexaenoic acid (DHA) isomer | MOL622013 | 6202762 | High | 5 |
| 1061 | Alanine isomer | MOL850013 | 8039084 | High | 3 |
| 1062 | Artemisinin | MOL901040 | 2581507 | High | 2 |
| 1063 | Fisetin | MOL338299 | 5930381 | High | 2 |
| 1064 | Artemisinin | MOL613863 | 1650577 | High | 3 |
| 1065 | Xanthohumol | MOL683218 | 13860987 | High | 4 |
| 1066 | Borneol | MOL798475 | 5738441 | High | 4 |
| 1067 | aesculetin | MOL192950 | 8043401 | High | 4 |
| 1068 | Parthenolide | MOL867075 | 4413291 | High | 4 |
| 1069 | Arginine | MOL408900 | 9684275 | High | 3 |
| 1070 | Verbenone isomer | MOL882228 | 14262724 | High | 2 |
| 1071 | Gamma-Linolenic acid | MOL994506 | 11197935 | High | 4 |
| 1072 | ethyl vanillin | MOL714342 | 14537952 | High | 3 |
| 1073 | Icariin | MOL795319 | 1315112 | High | 5 |
| 1074 | ZINC53300116 | MOL342286 | 238690 | High | 5 |
| 1075 | Withaferin A | MOL917098 | 11921434 | High | 5 |
| 1076 | Stevioside | MOL773525 | 7776010 | High | 4 |
| 1077 | Echinacoside | MOL339956 | 2234693 | High | 3 |
| 1078 | coumarin | MOL528855 | 14521820 | High | 3 |
| 1079 | Lupeol | MOL564575 | 11138811 | High | 4 |
| 1080 | Myricetin | MOL765180 | 11144503 | High | 2 |
| 1081 | Xanthohumol | MOL376608 | 3512815 | High | 5 |
| 1082 | Galangin | MOL250699 | 2566834 | High | 5 |
| 1083 | Isoeugenol | MOL448292 | 13017177 | High | 2 |
| 1084 | Lupeol | MOL665633 | 6210252 | High | 4 |
| 1085 | Yohimbine | MOL595548 | 13745712 | High | 3 |
| 1086 | Citronellol | MOL194374 | 1343941 | High | 3 |
| 1087 | Inositol | MOL603471 | 10945634 | High | 4 |
| 1088 | Withaferin A | MOL644519 | 2847081 | High | 3 |
| 1089 | Ergothioneine | MOL314160 | 15086014 | High | 4 |
| 1090 | Arginine | MOL654296 | 10496189 | High | 4 |
| 1091 | Stevioside | MOL675000 | 7895287 | High | 3 |
| 1092 | Choline | MOL594235 | 1203779 | High | 2 |
| 1093 | Scutellarin | MOL362355 | 12771247 | High | 2 |
| 1094 | Asparagine | MOL877417 | 8004939 | High | 3 |
| 1095 | Yohimbine | MOL864085 | 1021509 | High | 2 |
| 1096 | Arginine | MOL477794 | 7419360 | High | 5 |
| 1097 | Cedrol | MOL675082 | 5390937 | High | 5 |
| 1098 | Tyrosine | MOL154642 | 2027047 | High | 3 |
| 1099 | Creatine | MOL492289 | 2997820 | High | 3 |
| 1100 | Farnesol | MOL657591 | 14342265 | High | 3 |
| 1101 | Chrysin | MOL334790 | 1037358 | High | 2 |
| 1102 | Fisetin | MOL229886 | 13732417 | High | 4 |
| 1103 | Procyanidin B2 | MOL223224 | 10469378 | High | 2 |
| 1104 | vanillin | MOL965497 | 2527967 | High | 4 |
| 1105 | Galangin | MOL708663 | 9849660 | High | 4 |
| 1106 | Serine | MOL201711 | 5599427 | High | 4 |
| 1107 | Isoeugenol isomer | MOL551993 | 181523 | High | 3 |
| 1108 | vanillin | MOL135749 | 7267906 | High | 4 |
| 1109 | Glutamic acid | MOL113627 | 2034984 | High | 2 |
| 1110 | Gamma-Linolenic acid | MOL544031 | 5691830 | High | 4 |
| 1111 | caprylic acid | MOL828381 | 6584168 | High | 5 |
| 1112 | Docosahexaenoic acid (DHA) isomer | MOL604603 | 5880911 | High | 2 |
| 1113 | coumarin | MOL652385 | 10868368 | High | 2 |
| 1114 | Psoralen | MOL915304 | 4522984 | High | 3 |
| 1115 | Lupeol | MOL536650 | 4752470 | High | 3 |
| 1116 | Linalyl acetate | MOL289756 | 5773790 | High | 4 |
| 1117 | Saponin | MOL368793 | 13343443 | High | 2 |
| 1118 | Artemisinin | MOL321549 | 11205942 | High | 3 |
| 1119 | Cedrol | MOL956237 | 3469710 | High | 3 |
| 1120 | Parthenolide | MOL912627 | 10926349 | High | 3 |
| 1121 | Myricetin | MOL724918 | 11236258 | High | 4 |
| 1122 | Farnesol | MOL710435 | 12135945 | High | 4 |
| 1123 | Galangin | MOL465169 | 2518054 | High | 2 |
| 1124 | Diosgenin | MOL149112 | 4517402 | High | 4 |
| 1125 | Borneol | MOL853877 | 14610420 | High | 4 |
| 1126 | Proline | MOL182435 | 13311683 | High | 5 |
| 1127 | Zearalenone | MOL446230 | 709520 | High | 2 |
| 1128 | Borneol | MOL321449 | 2523351 | High | 5 |
| 1129 | Patchouli alcohol | MOL962257 | 2660280 | High | 2 |
| 1130 | Taxifolin | MOL477713 | 8112329 | High | 2 |
| 1131 | Procyanidin B2 | MOL374955 | 4715546 | High | 2 |
| 1132 | Nerolidol | MOL860889 | 3622091 | High | 2 |
| 1133 | Salicin | MOL769308 | 12937865 | High | 3 |
| 1134 | Withaferin A | MOL109143 | 6418755 | High | 3 |
| 1135 | Taxifolin | MOL489593 | 14969570 | High | 2 |
| 1136 | Terpinen-4-ol | MOL469364 | 8956754 | High | 3 |
| 1137 | Choline | MOL684213 | 11265438 | High | 5 |
| 1138 | Baicalin | MOL814291 | 9430789 | High | 5 |
| 1139 | Stevioside | MOL923890 | 14545125 | High | 5 |
| 1140 | Scutellarin | MOL219391 | 10125936 | High | 3 |
| 1141 | Eicosapentaenoic acid (EPA) | MOL741539 | 9551302 | High | 4 |
| 1142 | Asparagine | MOL343685 | 3312076 | High | 4 |
| 1143 | Xanthohumol | MOL818573 | 5178345 | High | 5 |
| 1144 | Gamma-Linolenic acid isomer | MOL941981 | 12351486 | High | 3 |
| 1145 | Myristic acid | MOL595976 | 12713788 | High | 2 |
| 1146 | caprylic acid | MOL278024 | 13268882 | High | 5 |
| 1147 | Stevioside | MOL325142 | 9939125 | High | 3 |
| 1148 | Cedrol | MOL197466 | 3409161 | High | 3 |
| 1149 | Procyanidin B2 isomer | MOL788807 | 1698780 | High | 5 |
| 1150 | Zearalenone | MOL620443 | 12047683 | High | 2 |
| 1151 | Serine | MOL321068 | 4426653 | High | 5 |
| 1152 | Myristic acid isomer | MOL757708 | 7956538 | High | 2 |
| 1153 | Chrysin | MOL526429 | 8946241 | High | 4 |
| 1154 | Terpinen-4-ol | MOL567711 | 1835002 | High | 5 |
| 1155 | Proline | MOL498590 | 12461718 | High | 5 |
| 1156 | Lecithin | MOL619061 | 13741605 | High | 2 |
| 1157 | Stearic acid | MOL156824 | 9628967 | High | 3 |
| 1158 | vanillin | MOL758387 | 14233354 | High | 2 |
| 1159 | Threonine | MOL274399 | 3417324 | High | 4 |
| 1160 | Icariin | MOL521163 | 12480925 | High | 4 |
| 1161 | Echinacoside | MOL458141 | 5030694 | High | 5 |
| 1162 | Parthenolide | MOL206661 | 1147061 | High | 3 |
| 1163 | Isoeugenol isomer | MOL373993 | 3459131 | High | 4 |
| 1164 | syringaldehyde | MOL421814 | 1534266 | High | 4 |
| 1165 | Verbenone isomer | MOL248795 | 14177070 | High | 3 |
| 1166 | Plumbagin | MOL854733 | 6515686 | High | 2 |
| 1167 | Stevioside isomer | MOL684368 | 2067120 | High | 4 |
| 1168 | vanillin | MOL159156 | 4740545 | High | 3 |
| 1169 | Alpha-Linolenic acid | MOL658834 | 3509185 | High | 3 |
| 1170 | Betaine | MOL721079 | 2785715 | High | 3 |
| 1171 | Glycine | MOL668725 | 5190317 | High | 5 |
| 1172 | Choline | MOL641251 | 3813559 | High | 2 |
| 1173 | Oleic acid | MOL552384 | 13886559 | High | 3 |
| 1174 | Borneol | MOL786999 | 1926772 | High | 4 |
| 1175 | Glycine | MOL804239 | 8028116 | High | 2 |
| 1176 | Diosgenin isomer | MOL450972 | 4587163 | High | 2 |
| 1177 | Linoleic acid | MOL769772 | 13940410 | High | 3 |
| 1178 | Patchouli alcohol | MOL411455 | 12936396 | High | 2 |
| 1179 | Chrysin | MOL519615 | 9713109 | High | 3 |
| 1180 | Matrine | MOL713682 | 730109 | High | 4 |
| 1181 | Artemisinin | MOL273250 | 14681723 | High | 4 |
| 1182 | Creatine isomer | MOL234675 | 6070085 | High | 2 |
| 1183 | Psoralen | MOL690188 | 2502954 | High | 5 |
| 1184 | Threonine | MOL202148 | 11691066 | High | 4 |
| 1185 | Tryptophan | MOL213506 | 11664779 | High | 2 |
| 1186 | Proline | MOL574575 | 2722541 | High | 2 |
| 1187 | Isoleucine | MOL624530 | 6408841 | High | 2 |
| 1188 | Plumbagin | MOL902673 | 9158142 | High | 3 |
| 1189 | Yohimbine | MOL643360 | 13556007 | High | 2 |
| 1190 | Borneol | MOL194012 | 8690169 | High | 4 |
| 1191 | Valine | MOL930789 | 1651889 | High | 3 |
| 1192 | coumarin | MOL953839 | 2445289 | High | 2 |
| 1193 | Glutathione | MOL687101 | 10120585 | High | 2 |
| 1194 | Alanine | MOL316208 | 12572906 | High | 4 |
| 1195 | Stevioside | MOL918974 | 13257289 | High | 5 |
| 1196 | Ellagic acid | MOL555858 | 4830640 | High | 2 |
| 1197 | Citronellol | MOL453305 | 12271378 | High | 5 |
| 1198 | vanillin isomer | MOL714705 | 13222715 | High | 3 |
| 1199 | Parthenolide | MOL127409 | 13645880 | High | 2 |
| 1200 | Tryptophan | MOL820885 | 6287672 | High | 5 |
| 1201 | Inositol | MOL691382 | 2569308 | High | 3 |
| 1202 | aesculetin | MOL542206 | 4427703 | High | 3 |
| 1203 | Matrine | MOL418440 | 1071916 | High | 2 |
| 1204 | Oleic acid | MOL891851 | 2285042 | High | 4 |
| 1205 | Fisetin isomer | MOL577880 | 14174617 | High | 4 |
| 1206 | Andrographolide | MOL332896 | 6016057 | High | 2 |
| 1207 | Terpinen-4-ol | MOL343683 | 8214187 | High | 4 |
| 1208 | caprylic acid | MOL507706 | 1735744 | High | 4 |
| 1209 | Stearic acid | MOL519547 | 13918894 | High | 3 |
| 1210 | Alanine | MOL768684 | 408794 | High | 2 |
| 1211 | coumarin | MOL400747 | 3168295 | High | 5 |
| 1212 | Salicin | MOL430882 | 5654190 | High | 4 |
| 1213 | Saponin | MOL636417 | 12019771 | High | 5 |
| 1214 | Parthenolide | MOL402386 | 5497277 | High | 3 |
| 1215 | Silymarin isomer | MOL573168 | 8207361 | High | 2 |
| 1216 | Farnesol | MOL851284 | 14993647 | High | 4 |
| 1217 | syringaldehyde | MOL545869 | 108696 | High | 2 |
| 1218 | Glutathione | MOL966134 | 7281585 | High | 2 |
| 1219 | Ergothioneine | MOL117090 | 14068893 | High | 4 |
| 1220 | Verbenone | MOL561616 | 12271580 | High | 4 |
| 1221 | scopoletin | MOL987992 | 12658331 | High | 3 |
| 1222 | Nerolidol | MOL420802 | 3247625 | High | 5 |
| 1223 | Colchicine | MOL909190 | 8833014 | High | 5 |
| 1224 | Betaine isomer | MOL291112 | 12800358 | High | 2 |
| 1225 | Arachidonic acid | MOL210293 | 14178649 | High | 2 |
| 1226 | Baicalin | MOL513954 | 4711403 | High | 5 |
| 1227 | Asparagine | MOL597222 | 6693840 | High | 5 |
| 1228 | Farnesol | MOL566273 | 13876366 | High | 2 |
| 1229 | Matrine | MOL634395 | 2649313 | High | 3 |
| 1230 | Lupeol | MOL420720 | 13158280 | High | 5 |
| 1231 | Proline isomer | MOL217277 | 5442389 | High | 2 |
| 1232 | Osthole | MOL809780 | 11032276 | High | 4 |
| 1233 | Tryptophan | MOL985409 | 10636600 | High | 2 |
| 1234 | Docosahexaenoic acid (DHA) | MOL819605 | 5622107 | High | 3 |
| 1235 | vanillin | MOL762102 | 2951005 | High | 5 |
| 1236 | Carnosic acid | MOL812727 | 980333 | High | 3 |
| 1237 | Stearic acid | MOL337906 | 1371763 | High | 3 |
| 1238 | Stearic acid isomer | MOL642707 | 9711752 | High | 3 |
| 1239 | Linoleic acid | MOL471058 | 2840486 | High | 5 |
| 1240 | Triptolide | MOL105805 | 6643454 | High | 4 |
| 1241 | coumarin | MOL292064 | 2619357 | High | 2 |
| 1242 | caproic acid isomer | MOL521574 | 4282198 | High | 5 |
| 1243 | ZINC55110686 | MOL417349 | 225385 | High | 3 |
| 1244 | Osthole | MOL390309 | 13914218 | High | 3 |
| 1245 | Creatine | MOL907339 | 7983662 | High | 3 |
| 1246 | Plumbagin | MOL894593 | 4327204 | High | 3 |
| 1247 | Salicin | MOL898759 | 3546564 | High | 2 |
| 1248 | Eicosapentaenoic acid (EPA) | MOL339790 | 4516399 | High | 5 |
| 1249 | Colchicine isomer | MOL566898 | 10522940 | High | 5 |
| 1250 | Baicalin | MOL393913 | 875900 | High | 3 |
| 1251 | Myricetin | MOL330471 | 9037889 | High | 5 |
| 1252 | Glutathione | MOL137062 | 14058048 | High | 5 |
| 1253 | Oleic acid | MOL981111 | 14094104 | High | 3 |
| 1254 | Glycine | MOL959395 | 12615716 | High | 2 |
| 1255 | Procyanidin B2 | MOL419440 | 4605165 | High | 4 |
| 1256 | Arachidonic acid | MOL964480 | 12431717 | High | 3 |
| 1257 | Leucine | MOL120498 | 2942437 | High | 5 |
| 1258 | Asparagine | MOL689278 | 3682375 | High | 5 |
| 1259 | syringaldehyde | MOL677281 | 7997974 | High | 3 |
| 1260 | Isoleucine | MOL195825 | 8577029 | High | 3 |
| 1261 | Myricetin | MOL773123 | 11466525 | High | 2 |
| 1262 | ZINC21162977 | MOL766291 | 4002511 | High | 2 |
| 1263 | Tryptophan | MOL553386 | 12843488 | High | 5 |
| 1264 | butyric acid | MOL933282 | 1801995 | High | 3 |
| 1265 | Arginine | MOL442202 | 6391436 | High | 4 |
| 1266 | Eicosapentaenoic acid (EPA) | MOL793480 | 11700635 | High | 3 |
| 1267 | Patchouli alcohol | MOL806244 | 6786123 | High | 4 |
| 1268 | Baicalin | MOL985282 | 4690548 | High | 4 |
| 1269 | caproic acid | MOL704077 | 10145703 | High | 2 |
| 1270 | Silymarin | MOL107866 | 14317006 | High | 3 |
| 1271 | ethyl vanillin | MOL144787 | 14763820 | High | 3 |
| 1272 | Serine | MOL722836 | 8169428 | High | 2 |
| 1273 | Diosgenin | MOL567619 | 10062762 | High | 2 |
| 1274 | Parthenolide | MOL913665 | 9169092 | High | 3 |
| 1275 | ethyl vanillin | MOL907967 | 7003052 | High | 4 |
| 1276 | Nerolidol | MOL185284 | 12574019 | High | 2 |
| 1277 | Borneol | MOL298587 | 9675922 | High | 3 |
| 1278 | Lauric acid | MOL507925 | 9859557 | High | 4 |
| 1279 | Verbenone | MOL893222 | 3519974 | High | 3 |
| 1280 | Serine | MOL901685 | 679577 | High | 3 |
| 1281 | Tyrosine isomer | MOL281710 | 8640635 | High | 5 |
| 1282 | Psoralen isomer | MOL508891 | 8078838 | High | 2 |
| 1283 | Sinomenine | MOL740958 | 7183366 | High | 4 |
| 1284 | Lauric acid | MOL949257 | 14353207 | High | 3 |
| 1285 | capric acid | MOL987740 | 2287581 | High | 3 |
| 1286 | Alanine | MOL207085 | 12768477 | High | 3 |
| 1287 | Lauric acid | MOL871815 | 10811436 | High | 3 |
| 1288 | syringaldehyde | MOL892176 | 194017 | High | 5 |
| 1289 | Ergothioneine | MOL499534 | 6269170 | High | 2 |
| 1290 | Baicalin | MOL844251 | 2190269 | High | 3 |
| 1291 | Vincristine | MOL549265 | 14691372 | High | 5 |
| 1292 | Gamma-Linolenic acid | MOL708789 | 14071173 | High | 2 |
| 1293 | Tryptophan isomer | MOL483418 | 2040665 | High | 3 |
| 1294 | Celastrol isomer | MOL896700 | 12834561 | High | 3 |
| 1295 | Diosgenin | MOL430310 | 7443604 | High | 2 |
| 1296 | Yohimbine | MOL699359 | 3419482 | High | 3 |
| 1297 | Chrysin | MOL763974 | 6194294 | High | 2 |
| 1298 | Terpinen-4-ol | MOL698431 | 12041769 | High | 2 |
| 1299 | Alpha-Bisabolol | MOL590932 | 12435039 | High | 2 |
| 1300 | Cedrol | MOL851862 | 982930 | High | 5 |
| 1301 | Isoeugenol | MOL123177 | 7990089 | High | 4 |
| 1302 | Galangin | MOL833404 | 9300339 | High | 2 |
| 1303 | Leucine | MOL924488 | 11985019 | High | 5 |
| 1304 | caprylic acid | MOL354905 | 9764998 | High | 5 |
| 1305 | Icariin | MOL627607 | 2771880 | High | 2 |
| 1306 | Yohimbine | MOL180560 | 7587447 | High | 3 |
| 1307 | Triptolide isomer | MOL344478 | 9696212 | High | 3 |
| 1308 | Farnesol isomer | MOL687754 | 2827365 | High | 5 |
| 1309 | Silymarin | MOL888431 | 7282889 | High | 2 |
| 1310 | Isoleucine | MOL868170 | 2669282 | High | 2 |
| 1311 | Lecithin | MOL737672 | 5721553 | High | 4 |
| 1312 | Threonine | MOL746913 | 9658965 | High | 5 |
| 1313 | Yohimbine isomer | MOL799272 | 7942891 | High | 2 |
| 1314 | Myristic acid | MOL246542 | 12920317 | High | 2 |
| 1315 | Arachidonic acid | MOL236482 | 366940 | High | 4 |
| 1316 | Choline | MOL396315 | 14050691 | High | 2 |
| 1317 | Ergothioneine isomer | MOL136854 | 13580652 | High | 4 |
| 1318 | ethyl vanillin | MOL436033 | 13501400 | High | 5 |
| 1319 | coumarin isomer | MOL178440 | 11918748 | High | 4 |
| 1320 | Vincristine | MOL600797 | 3757878 | High | 3 |
| 1321 | capric acid | MOL803682 | 10620990 | High | 4 |
| 1322 | Parthenolide | MOL494697 | 5183040 | High | 4 |
| 1323 | Triptolide | MOL464586 | 2122918 | High | 5 |
| 1324 | Scutellarin | MOL621498 | 10878506 | High | 4 |
| 1325 | Baicalin | MOL970469 | 8794133 | High | 5 |
| 1326 | Hesperetin | MOL591418 | 12229650 | High | 2 |
| 1327 | scopoletin | MOL869461 | 12097881 | High | 4 |
| 1328 | Isoeugenol | MOL684123 | 6598289 | High | 2 |
| 1329 | Galangin | MOL688618 | 2816175 | High | 5 |
| 1330 | Patchouli alcohol | MOL689823 | 11251058 | High | 4 |
| 1331 | ethyl vanillin | MOL480015 | 15032199 | High | 5 |
| 1332 | Glutathione | MOL679850 | 12339290 | High | 5 |
| 1333 | capric acid | MOL977400 | 5044336 | High | 5 |
| 1334 | Phloretin | MOL187505 | 927152 | High | 3 |
| 1335 | Hesperetin | MOL311131 | 3042700 | High | 2 |
| 1336 | Silymarin | MOL138488 | 7086309 | High | 2 |
| 1337 | Geranyl acetate | MOL391155 | 6158918 | High | 4 |
| 1338 | Fisetin | MOL968697 | 4643608 | High | 4 |
| 1339 | Lecithin isomer | MOL417646 | 7456598 | High | 4 |
| 1340 | Galangin | MOL322668 | 12790468 | High | 4 |
| 1341 | Proline | MOL414754 | 7884774 | High | 2 |
| 1342 | Farnesol isomer | MOL228732 | 1546976 | High | 4 |
| 1343 | Ellagic acid | MOL525352 | 6244547 | High | 4 |
| 1344 | Ellagic acid | MOL859913 | 12368919 | High | 3 |
| 1345 | Threonine | MOL154403 | 11779399 | High | 5 |
| 1346 | butyric acid isomer | MOL927877 | 13177420 | High | 5 |
| 1347 | Colchicine | MOL937092 | 5159066 | High | 2 |
| 1348 | Triptolide isomer | MOL749929 | 4490853 | High | 3 |
| 1349 | Asparagine | MOL205690 | 3862997 | High | 5 |
| 1350 | Glutamine | MOL269374 | 14880931 | High | 5 |
| 1351 | Taxifolin | MOL141564 | 10295764 | High | 4 |
| 1352 | Xanthohumol | MOL947916 | 12453301 | High | 4 |
| 1353 | Procyanidin B2 | MOL746059 | 10599152 | High | 5 |
| 1354 | Arginine isomer | MOL971623 | 4154756 | High | 2 |
| 1355 | Withaferin A | MOL820534 | 11361512 | High | 2 |
| 1356 | Asparagine | MOL800493 | 12385632 | High | 2 |
| 1357 | Artemisinin | MOL617565 | 8983090 | High | 2 |
| 1358 | Baicalin | MOL248961 | 2900810 | High | 5 |
| 1359 | Echinacoside | MOL517403 | 3445628 | High | 3 |
| 1360 | Lecithin | MOL879462 | 14450717 | High | 4 |
| 1361 | Alpha-Linolenic acid | MOL675787 | 8544917 | High | 5 |
| 1362 | Threonine isomer | MOL862546 | 4960714 | High | 5 |
| 1363 | Psoralen | MOL979870 | 5563751 | High | 4 |
| 1364 | Glutamine | MOL868744 | 10979560 | High | 2 |
| 1365 | Nerolidol | MOL458187 | 3184690 | High | 4 |
| 1366 | vanillin isomer | MOL919959 | 9850315 | High | 3 |
| 1367 | Lupeol | MOL653074 | 4092939 | High | 2 |
| 1368 | Psoralen | MOL494447 | 7249846 | High | 2 |
| 1369 | Gamma-Linolenic acid | MOL520683 | 8749910 | High | 5 |
| 1370 | Stearic acid isomer | MOL571067 | 14322178 | High | 5 |
| 1371 | capric acid | MOL596967 | 7207010 | High | 3 |
| 1372 | Lauric acid | MOL251682 | 5952340 | High | 4 |
| 1373 | Vincristine | MOL857134 | 2630649 | High | 3 |
| 1374 | ethyl vanillin | MOL258751 | 13062785 | High | 3 |
| 1375 | Tyrosine | MOL220189 | 8840213 | High | 4 |
| 1376 | Saponin | MOL580007 | 4134392 | High | 2 |
| 1377 | vanillin | MOL321022 | 5597141 | High | 3 |
| 1378 | Stevioside | MOL655042 | 10036189 | High | 5 |
| 1379 | Eicosapentaenoic acid (EPA) isomer | MOL434068 | 6391929 | High | 3 |
| 1380 | Artemisinin | MOL803885 | 10377951 | High | 4 |
| 1381 | syringaldehyde | MOL181309 | 3590274 | High | 5 |
| 1382 | Osthole | MOL160481 | 6330507 | High | 4 |
| 1383 | Andrographolide | MOL660679 | 4327085 | High | 4 |
| 1384 | Myristic acid | MOL390472 | 10024760 | High | 3 |
| 1385 | Saponin | MOL355796 | 9911001 | High | 3 |
| 1386 | Chrysin | MOL139840 | 11586467 | High | 2 |
| 1387 | Cedrol | MOL151251 | 4087332 | High | 2 |
| 1388 | Patchouli alcohol | MOL272712 | 14230726 | High | 5 |
| 1389 | Procyanidin B2 isomer | MOL404941 | 13096492 | High | 2 |
| 1390 | Carnosic acid isomer | MOL780549 | 3142356 | High | 2 |
| 1391 | Isoeugenol | MOL776170 | 13862870 | High | 4 |
| 1392 | Yohimbine | MOL771471 | 9095709 | High | 2 |
| 1393 | Docosahexaenoic acid (DHA) | MOL703887 | 3190174 | High | 2 |
| 1394 | Yohimbine | MOL127511 | 13177315 | High | 3 |
| 1395 | Patchouli alcohol | MOL128056 | 12367206 | High | 5 |
| 1396 | Arginine | MOL663698 | 4819524 | High | 2 |
| 1397 | Asparagine | MOL572777 | 5947634 | High | 4 |
| 1398 | Silymarin | MOL219285 | 3748300 | High | 4 |
| 1399 | Cedrol | MOL622750 | 13321226 | High | 3 |
| 1400 | Linoleic acid | MOL601329 | 1001222 | High | 5 |
| 1401 | Chrysin | MOL631148 | 3176927 | High | 5 |
| 1402 | Borneol | MOL742987 | 5712992 | High | 5 |
| 1403 | Isoleucine | MOL829716 | 6899301 | High | 2 |
| 1404 | Patchouli alcohol | MOL351943 | 5370737 | High | 3 |
| 1405 | Docosahexaenoic acid (DHA) | MOL158127 | 10386795 | High | 5 |
| 1406 | Glutamic acid | MOL456472 | 9608085 | High | 5 |
| 1407 | Glycine | MOL393822 | 3005877 | High | 5 |
| 1408 | Alpha-Bisabolol | MOL143190 | 9872959 | High | 3 |
| 1409 | Procyanidin B2 | MOL849917 | 9339288 | High | 2 |
| 1410 | Linoleic acid | MOL137063 | 11981046 | High | 4 |
| 1411 | Zearalenone | MOL960271 | 13732393 | High | 2 |
| 1412 | syringaldehyde | MOL187894 | 1861521 | High | 3 |
| 1413 | Alpha-Bisabolol | MOL332427 | 6756339 | High | 5 |
| 1414 | Glutamine | MOL347738 | 13302956 | High | 2 |
| 1415 | Linoleic acid | MOL620051 | 6420150 | High | 5 |
| 1416 | butyric acid | MOL710430 | 504587 | High | 3 |
| 1417 | Proline | MOL910465 | 6511504 | High | 3 |
| 1418 | Galangin | MOL409749 | 7768820 | High | 5 |
| 1419 | Proline | MOL367726 | 2797990 | High | 3 |
| 1420 | Arginine | MOL327672 | 1532114 | High | 3 |
| 1421 | Triptolide | MOL880831 | 13460207 | High | 3 |
| 1422 | Verbenone isomer | MOL762288 | 14678932 | High | 3 |
| 1423 | Tryptophan | MOL482747 | 14689229 | High | 4 |
| 1424 | Leucine | MOL105953 | 3770457 | High | 5 |
| 1425 | Glutathione | MOL639762 | 1847179 | High | 5 |
| 1426 | Inositol | MOL610139 | 2023391 | High | 4 |
| 1427 | ethyl vanillin | MOL993627 | 6369150 | High | 5 |
| 1428 | Leucine | MOL521611 | 2870258 | High | 5 |
| 1429 | Celastrol | MOL367221 | 3368065 | High | 3 |
| 1430 | Arginine | MOL376058 | 2200749 | High | 5 |
| 1431 | Procyanidin B2 | MOL356907 | 13742003 | High | 5 |
| 1432 | Isoleucine | MOL129746 | 5385788 | High | 4 |
| 1433 | butyric acid | MOL273435 | 12486313 | High | 2 |
| 1434 | Yohimbine | MOL696968 | 12682034 | High | 4 |
| 1435 | Yohimbine | MOL626589 | 3518488 | High | 5 |
| 1436 | Lauric acid | MOL374719 | 11936659 | High | 3 |
| 1437 | Tryptophan | MOL858731 | 1113103 | High | 5 |
| 1438 | Isoeugenol | MOL717694 | 890262 | High | 4 |
| 1439 | butyric acid | MOL915468 | 3162170 | High | 2 |
| 1440 | Arginine | MOL159289 | 3319533 | High | 4 |
| 1441 | Glycine isomer | MOL241231 | 12668595 | High | 3 |
| 1442 | Withaferin A | MOL164460 | 2792897 | High | 4 |
| 1443 | Sinomenine | MOL989038 | 12955848 | High | 3 |
| 1444 | Glutathione | MOL845387 | 8274477 | High | 3 |
| 1445 | Stevioside | MOL138335 | 4829599 | High | 2 |
| 1446 | Geranyl acetate | MOL339331 | 2942658 | High | 2 |
| 1447 | syringaldehyde isomer | MOL646846 | 11856187 | High | 4 |
| 1448 | Glutathione isomer | MOL542270 | 8138160 | High | 3 |
| 1449 | Verbenone | MOL145354 | 3294024 | High | 4 |
| 1450 | Glutamine | MOL452597 | 5608900 | High | 4 |
| 1451 | coumarin isomer | MOL276403 | 3176709 | High | 5 |
| 1452 | butyric acid | MOL120917 | 3691375 | High | 3 |
| 1453 | Plumbagin | MOL919093 | 10239258 | High | 4 |
| 1454 | Threonine isomer | MOL581601 | 4738711 | High | 5 |
| 1455 | Glutamine isomer | MOL277456 | 9485384 | High | 3 |
| 1456 | ethyl vanillin | MOL267811 | 13157991 | High | 3 |
| 1457 | Alanine | MOL812498 | 5615736 | High | 4 |
| 1458 | Valine isomer | MOL311276 | 976908 | High | 5 |
| 1459 | Choline | MOL930405 | 9409108 | High | 4 |
| 1460 | Gamma-Linolenic acid | MOL360291 | 1582089 | High | 4 |
| 1461 | Lupeol | MOL793886 | 13687104 | High | 3 |
| 1462 | ZINC81676771 | MOL678492 | 5824953 | High | 5 |
| 1463 | Taxifolin | MOL558744 | 12557972 | High | 4 |
| 1464 | Valine | MOL604517 | 4675728 | High | 3 |
| 1465 | Geranyl acetate | MOL557551 | 10930378 | High | 4 |
| 1466 | syringaldehyde | MOL544086 | 11974146 | High | 3 |
| 1467 | Colchicine | MOL570921 | 12375865 | High | 5 |
| 1468 | Taxifolin | MOL124969 | 14552654 | High | 2 |
| 1469 | Ellagic acid | MOL142535 | 10991335 | High | 4 |
| 1470 | Galangin | MOL864099 | 118519 | High | 3 |
| 1471 | Diosgenin | MOL596649 | 213499 | High | 4 |
| 1472 | Glutathione | MOL419099 | 861561 | High | 3 |
| 1473 | Procyanidin B2 | MOL998821 | 7647338 | High | 2 |
| 1474 | vanillin | MOL615827 | 11165817 | High | 4 |
| 1475 | Glutamic acid | MOL321635 | 10547051 | High | 5 |
| 1476 | Lauric acid | MOL769995 | 4804606 | High | 3 |
| 1477 | Inositol | MOL249794 | 10213751 | High | 5 |
| 1478 | caproic acid | MOL288266 | 6740986 | High | 5 |
| 1479 | Taxifolin | MOL836006 | 5799631 | High | 2 |
| 1480 | Terpinen-4-ol | MOL669218 | 11198955 | High | 4 |
| 1481 | Carnosic acid | MOL135460 | 1837705 | High | 2 |
| 1482 | Echinacoside | MOL739365 | 6792916 | High | 5 |
| 1483 | Ellagic acid | MOL251447 | 7223641 | High | 4 |
| 1484 | syringaldehyde isomer | MOL861720 | 2477261 | High | 3 |
| 1485 | Ellagic acid | MOL309132 | 5663244 | High | 3 |
| 1486 | Valine | MOL755304 | 12713144 | High | 4 |
| 1487 | scopoletin | MOL817955 | 10936289 | High | 5 |
| 1488 | Triptolide | MOL530221 | 2316428 | High | 4 |
| 1489 | Arginine | MOL157682 | 4816993 | High | 5 |
| 1490 | Echinacoside | MOL856287 | 1245621 | High | 2 |
| 1491 | Vincristine | MOL125552 | 1393756 | High | 5 |
| 1492 | Tryptophan isomer | MOL772691 | 1996445 | High | 2 |
| 1493 | syringaldehyde | MOL278651 | 2362735 | High | 5 |
| 1494 | ethyl vanillin | MOL585559 | 10592769 | High | 3 |
| 1495 | Salicin | MOL994030 | 15013197 | High | 2 |
| 1496 | Threonine | MOL920924 | 14709917 | High | 5 |
| 1497 | Verbenone | MOL558292 | 991226 | High | 5 |
| 1498 | Tyrosine | MOL898720 | 12548697 | High | 5 |
| 1499 | Xanthohumol | MOL236071 | 8483958 | High | 2 |
| 1500 | Celastrol | MOL175577 | 10800047 | High | 5 |
| 1501 | syringaldehyde | MOL441098 | 11850324 | High | 3 |
| 1502 | Eicosapentaenoic acid (EPA) | MOL796470 | 5530714 | High | 2 |
| 1503 | Geranyl acetate isomer | MOL314605 | 6345297 | High | 4 |
| 1504 | Matrine | MOL858581 | 5225687 | High | 2 |
| 1505 | Tyrosine | MOL117502 | 3651283 | High | 4 |
| 1506 | Saponin | MOL599684 | 9390436 | High | 5 |
| 1507 | Alpha-Bisabolol | MOL917463 | 7193412 | High | 3 |
| 1508 | Sinomenine | MOL292478 | 938332 | High | 4 |
| 1509 | capric acid | MOL630479 | 7801867 | High | 3 |
| 1510 | Patchouli alcohol | MOL212818 | 1882274 | High | 4 |
| 1511 | Stevioside | MOL732554 | 167265 | High | 5 |
| 1512 | Myristic acid | MOL270697 | 7484760 | High | 3 |
| 1513 | Isoleucine isomer | MOL360923 | 8482016 | High | 5 |
| 1514 | Ergothioneine | MOL232554 | 10922304 | High | 2 |
| 1515 | Alanine | MOL683158 | 5769445 | High | 4 |
| 1516 | Yohimbine | MOL651215 | 231038 | High | 3 |
| 1517 | Inositol | MOL702868 | 12908022 | High | 3 |
| 1518 | Serine | MOL700961 | 14908108 | High | 5 |
| 1519 | Arginine | MOL771401 | 2481639 | High | 5 |
| 1520 | Lecithin | MOL759894 | 13765337 | High | 3 |
| 1521 | Baicalin | MOL677730 | 7290343 | High | 2 |
| 1522 | Linalyl acetate | MOL209664 | 3097688 | High | 5 |
| 1523 | Vincristine | MOL655512 | 5900613 | High | 4 |
| 1524 | Carnosic acid | MOL882951 | 9039072 | High | 3 |
| 1525 | Taxifolin | MOL707407 | 3153909 | High | 2 |
| 1526 | Saponin | MOL565258 | 5789128 | High | 5 |
| 1527 | Betaine | MOL749724 | 3426332 | High | 5 |
| 1528 | caprylic acid | MOL547380 | 6894176 | High | 4 |
| 1529 | Taxifolin | MOL116396 | 4455979 | High | 5 |
| 1530 | vanillin | MOL640573 | 9809723 | High | 3 |
| 1531 | butyric acid | MOL794282 | 3267552 | High | 2 |
| 1532 | Chrysin | MOL506456 | 7198800 | High | 2 |
| 1533 | Triptolide | MOL911997 | 5407616 | High | 2 |
| 1534 | Alanine | MOL632532 | 2703567 | High | 2 |
| 1535 | caprylic acid | MOL167199 | 6920287 | High | 5 |
| 1536 | caprylic acid | MOL237504 | 8182410 | High | 5 |
| 1537 | Silymarin | MOL648366 | 6371128 | High | 5 |
| 1538 | Choline | MOL500271 | 4256852 | High | 2 |
| 1539 | Carnosic acid | MOL797477 | 6458568 | High | 5 |
| 1540 | Alanine | MOL323317 | 7935409 | High | 2 |
| 1541 | Lecithin | MOL732249 | 11285668 | High | 2 |
| 1542 | Isoleucine | MOL635668 | 2090374 | High | 2 |
| 1543 | Linoleic acid | MOL303676 | 13590131 | High | 3 |
| 1544 | Serine | MOL908827 | 1663140 | High | 4 |
| 1545 | Alpha-Linolenic acid | MOL338518 | 7426966 | High | 5 |
